# QM/MM Benchmarking of Cyanobacteriochrome Slr1393g3 Absorption Spectra

**DOI:** 10.3390/molecules24091720

**Published:** 2019-05-03

**Authors:** Christian Wiebeler, Igor Schapiro

**Affiliations:** Fritz Haber Center for Molecular Dynamics Research, Institute of Chemistry, The Hebrew University of Jerusalem, Jerusalem 91904, Israel; chwie@mail.upb.de

**Keywords:** phytochrome, cyanobacteriochrome, Slr1393g3, spectral tuning, QM/MM, molecular dynamics, photochemistry, excited states

## Abstract

Cyanobacteriochromes are compact and spectrally diverse photoreceptor proteins that are promising candidates for biotechnological applications. Computational studies can contribute to an understanding at a molecular level of their wide spectral tuning and diversity. In this contribution, we benchmark methods to model a 110 nm shift in the UV/Vis absorption spectrum from a red- to a green-absorbing form of the cyanobacteriochrome Slr1393g3. Based on an assessment of semiempirical methods to describe the chromophore geometries of both forms in vacuo, we find that DFTB2+D leads to structures that are the closest to the reference method. The benchmark of the excited state calculations is based on snapshots from quantum mechanics/molecular mechanics molecular dynamics simulations. In our case, the methods RI-ADC(2) and sTD-DFT based on CAM-B3LYP ground state calculations perform the best, whereas no functional can be recommended to simulate the absorption spectra of both forms with time-dependent density functional theory. Furthermore, the difference in absorption for the lowest energy absorption maxima of both forms can already be modelled with optimized structures, but sampling is required to improve the shape of the absorption bands of both forms, in particular for the second band. This benchmark study can guide further computational studies, as it assesses essential components of a protocol to model the spectral tuning of both cyanobacteriochromes and the related phytochromes.

## 1. Introduction

**Scheme 1:**
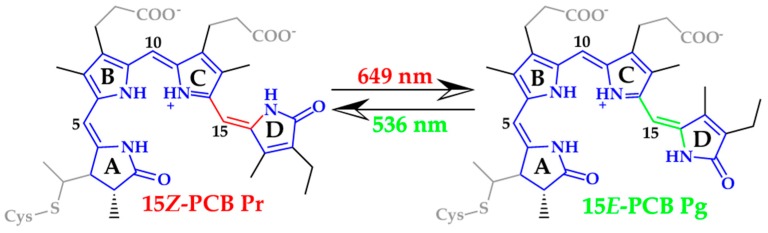
Schematic representation of the PCB structures in the red- and green-absorbing forms. The differences between the two forms are highlighted in red and green for P_r_ and P_g_, respectively. The wavelengths refer to the lowest energy absorption maxima of the measured spectra [13,14]. The QM region consisting of 42 atoms (QM42) is shown in blue, whereas the atoms of the QM region with 66 atoms (QM66) are shown accentuated in black.

Cyanobacteriochromes (CBCR) are a new family of photoreceptor proteins recently discovered in cyanobacteria [1]. They are photosensory proteins that allow the bacteria to adjust their lifestyle in response to the environmental illumination conditions [2]. Similar to the related canonical plant and bacterial phytochromes, CBCRs bind a bilin chromophore and they are photochromic, i.e., they can be photoswitched between two differently absorbing states [3]. Contrary to canonical phytochromes, where absorption is switched between the red and far-red light-absorbing form, CBCRs are spectrally diverse with color detection from near-ultraviolet to near-infrared (NIR), and a single cGMP-phosphodiesterase/adenylate cyclase/FhlA (GAF) domain is sufficient for chromophore attachment and complete photochemistry [4]. The wide spectral tuning of CBCRs, for which four distinct mechanisms have been identified so far [5], their compactness and reversible photochemistry allow to tailor their spectroscopic properties and make them ideal candidates for biotechnological applications, e.g., optogenetics and design of NIR fluorescent proteins (FP) for bioimaging [6,7]. Regarding the latter, first studies were dedicated to the development of NIR FPs based on phytochromes [8,9,10], but recently also a NIR FP based on a CBCR was designed [11]. However, to tailor CBCRs for specific applications, a molecular level understanding of the spectral tuning in these spectrally diverse proteins is required. In our recent publication, we could model the difference in position of the lowest energy absorption maxima between the photoproduct and the dark state, denoted as photoproduct tuning, of the prototypical red/green CBCR Slr1393g3 [12]. This CBCR harbors a phycocyanobilin (PCB) chromophore and exhibits a red-absorbing dark state (λ_max_ = 649 nm) and a green-absorbing photoproduct (λ_max_ = 536 nm) resulting in a photoproduct tuning of more than 100 nm, see Scheme 1. Therefore, the lowest energy absorption maxima of both forms are found in two different regions of the visible spectrum providing a model system to assess the quality of the quantum chemical methods to model the photoproduct tuning of a specific CBCR family and phytochromes in general.

Computational studies of CBCR proteins are limited to two investigations of AnPixJg2, a red/green CBCR. Both are based on its crystal structure as a starting point [15]. The first study by Velazquez-Escobar et al. employed classical molecular dynamics (MD) simulations to study both P_r_ and P_g_ forms [16]. For the latter, the PCB chromophore was isomerized manually in the P_r_ structure to mimic the photoproduct. In the second study by Scarbath-Evers et al., the P_r_ form was simulated with classical MD for 1 μs [17]. In both studies, no attempts were reported to simulate ultraviolet/visible (UV/Vis) absorption spectra. However, pure quantum mechanical (QM) calculations for the excited states of PCB that are related to investigations on CBCRs have been performed. These studies either used model compounds built from scratch and optimized in vacuo to mimic PCB in CBCRs [18,19] or the required atomic positions were extracted from crystallized protein structures. In case of the latter, typically only PCB without relevant, adjacent amino acids was extracted [20,21,22,23] with the exception of one study, where in addition also the conserved aspartate and some solvent molecules were included [20]. In all those studies, time-dependent density functional theory (TD-DFT) was employed for the excited state calculations. Based on calculated absorption and circular dichroism spectra conclusions about possible PCB conformations in the protein could be drawn [18,19,20,21,23]. Matute et al. found that excited state calculations based on X-ray geometries of PCB from the cyanobacterial phytochrome Cph1 yield better agreement with experimental absorption spectra than from freely relaxed PCB structures in vacuo [21]. Therefore and to the best of our knowledge, our recently published study on the photoproduct tuning of the red/green CBCR Slr1393g3 was the first one to combine the treatment of a CBCR protein via quantum mechanics/molecular mechanics (QM/MM) with the simulation of absorption spectra based on snapshots extracted from QM/MM MD [12]. In that study, we have put special emphasis on describing the difference in absorption between the dark state P_r_ and the photoproduct P_g_, as our simulations were based on crystal structures for both forms from the same protein [24].

Many more computational investigations were performed for canonical phytochromes than for CBCRs, therefore we do not attempt to give an exhaustive overview. Nonetheless, the majority of studies on phytochromes can be divided again into two categories: on the one hand, there are simulations of phytochrome proteins without determination of UV/Vis absorption spectra, but with an emphasis on structural elucidation and vibrational characterization. For this purpose, classical MD simulations were performed by several groups [25,26,27,28]. In addition, Mroginski and coworkers also employed QM/MM simulations [29,30,31] and some recent publications based on this approach can be found in references [32,33,34,35,36,37]. Recently a QM/MM approach was also applied to investigate the covalent binding of the biliverdin (BV) chromophore to phytochrome domains [38] in the NIR FP miRFP670, which was also investigated experimentally [39,40,41]. On the other hand, excitation energies for tetrapyrrole chromophores found in phytochromes were calculated in the gas phase or in implicit solvent. In particular, Durbeej et al. investigated phytochromobilin and reported among others that not including the thioether linkage, the propionate side chains and the methyl groups has only a small influence on excitation energies and oscillator strengths [42,43]. This was later extended by a benchmark study of locked bilin chromophores, where it was found that TD-DFT with pure generalized gradient approximation (GGA) functionals is a particularly viable choice of methodology for calculating absorption and emission maxima of bilin chromophores [44]. Furthermore, Matute et al. employed TD-DFT to predict the chromophore conformations, when they are bound to the proteins [21,45]. Also the photochemistry of related chromophore models was investigated by several groups [46,47,48,49] and more details on this topic, which involves an ultrafast double-bond isomerization, can be found in a recent review [50].

In case of phytochromes, also QM/MM-based methodology was applied to calculate excited states and to investigate their photochemistry [51,52,53,54,55], thereby connecting the two aforementioned categories. The initial study by Falklöf and Durbeej investigated the red light-absorbing form of the bacteriophytochrome from *Deinococcus radiodurans* (DrBphP) based on optimized structures and it was reported that pure GGA functionals reproduce the experimental Q-band maximum, i.e., lowest energy absorption maximum, in the red region with smaller errors than both global and range-separated hybrid functionals [51]. Similar conclusions were also reached by Nemukhin and coworkers studying structures and excitation energies for BV-binding domains in infrared fluorescent proteins [54] and by Modi et al. investigating both the red light and far-red light-absorbing forms of DrBphP to determine the protonation states of the chromophore in the corresponding forms [55]. In addition to TD-DFT calculations, both studies report computations with the extended multi-configuration quasi-degenerate perturbation theory to second order (XMCQDPT2) method [56] and in the investigation by Modi et al. structures were sampled directly from classical MD instead of using optimized structures. 

In the present benchmark study, we examine essential components in detail that are part of our protocol to model the photoproduct tuning in CBCRs and phytochromes, which was derived based on our previous works on Slr1393g3 [12]. Firstly, we assess the accuracy of semiempirical quantum mechanical (SQM) methods for the description of truncated PCB geometries in vacuo by comparing them with ab initio resolution of identity (RI) approximate coupled cluster singles and doubles (CC2) reference geometries, as SQM methods present an efficient approach to sample sufficient structures for excited state calculations. Secondly, we benchmark methods for these calculations with a focus on the description of the photoproduct tuning and the geometries for this comparison are taken from SQM/MM MD trajectories. Thirdly, we compare absorption spectra obtained from SQM/MM and QM/MM optimized structures to check how well the protein-embedded PCB geometries are described semiempirically. Finally, we discuss differences in absorption spectra, when they are determined either from optimized structures or averaged over different numbers of snapshots within one consistent approach to assess the importance of sampling.

## 2. Results

### 2.1. Chromophore Structure Optimizations with Semiempirical Methods

The truncated PCB geometries containing 42 atoms (see the blue parts in Scheme 1) were optimized in vacuo for both forms. The performance of SQM methods as implemented in the AMBER software package [57,58,59] is compared with corresponding RI-CC2/cc-pVDZ [60,61,62,63] optimized structures using Turbomole 7.0 [64,65]. The similarity between the obtained structures is evaluated based on the root mean square deviations (RMSD) of the aligned geometries. First, we note that the RMSD values between SQM methods and RI-CC2 are in general smaller for the P_r_ than for the P_g_ form. Furthermore, modified neglect of diatomic overlap (MNDO) [66], parameterized model 3 (PM3) [67] and PM3 with pairwise distance directed Gaussian method (PM3-PDDG) [68] exhibit RMSD values of around 1 Å for P_r_ and of at least 1.26 Å for P_g_, see Table S1. This also holds for parameterized model 6 (PM6) [69], but including corrections for dispersion and hydrogen bonding (DH+) [70,71] improves the agreement, in particular for P_g_, see Table S2. The same trend is observed in case of the Austin model 1 (AM1) methods [72], where the revised model AM1/d [73] exhibits a performance between the original AM1 method and its dispersion corrected version AM1-D. Again the best results are obtained when both dispersion and hydrogen bond corrections [70,71] are employed, see Table S3. Overall, AM1-DH+ and Recife model 1 (RM1) [74] are the two best performing semiempirical methods based on Hartree-Fock with RMSD values of around 0.66 Å (P_r_) and 1.00 Å (P_g_), followed by the PM6-DH+ method with RMSD values of 0.76 Å (P_r_) and 1.10 Å (P_g_). 

In addition, we performed optimizations with density functional tight binding (DFTB) approach [75,76]. For this family of SQM methods, DFTB2 with mio-1-1 parameters [77,78] and DFTB3 [79] with 3ob-3-1 parameters [80,81] are implemented in the AMBER software suite. For the truncated chromophore, the former method is found to yield structures that are closer to the RI-CC2 reference for both forms, see Table S4. The RMSD value of 0.79 Å for P_r_ is similar to the results from PM6-DH, whereas the value of 0.94 Å for P_g_ is already lower than in case of the Hartree-Fock based methods. Furthermore, using an empirical dispersion correction (+D) [82] in combination with DFTB2 results in further improvements: the RMSD values of 0.58 Å (P_r_) and 0.23 Å (P_g_) are for each form the lowest among all evaluated methods, see Table 1. However, no dispersion correction, e.g., Grimme′s dispersion correction D3 [83], is available in the current implementation of DFTB3 in the AMBER software package. Hence, we find DFTB2+D to be the most promising SQM method for the description of the PCB chromophore based on the assessment of in vacuo optimized geometries. 

**Figure 1:**
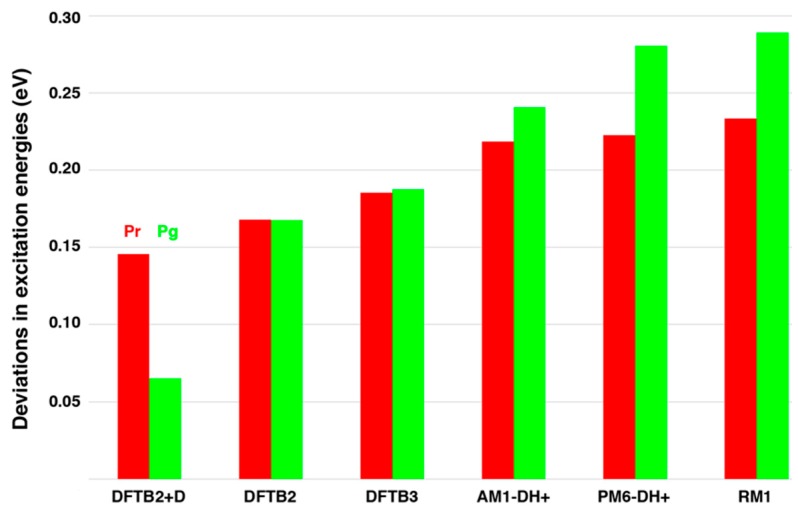
Deviations of the excitation energies for the first excited state based on differently optimized structures for QM42. The energies are calculated via the RI-CC2/cc-pVDZ level of theory for P_g_ (green) and P_r_ (red). The reference values from RI-CC2/cc-pVDZ optimizations are 2.01 eV and 2.00 eV for P_r_ and P_g_, respectively, see also Tables S5 and S6.

This is also corroborated by the calculation of excitation energies with RI-CC2 based on the differently optimized structures. The first ten excited states from these calculations are shown in Tables S5 and S6 for P_r_ and P_g_, respectively. In addition, the deviations in excitation energies from the RI-CC2 optimized reference structures for the first excited state, which is the origin of the lowest energy absorption band, are shown in Figure 1. Overall the excitation energies from the SQM optimized geometries are higher than those from RI-CC2. The smallest differences are found for DFTB2+D, therefore the good agreement for the P_g_ form could have already been expected based on its low RMSD value. This method is followed by DFTB2 and DFTB3 optimized structures, which exhibit RI-CC2 excitation energies somewhat lower than in case of the SQM methods based on Hartree-Fock, even though the RMSD values are comparable. The deviations for the three Hartree-Fock based methods are nearly the same for P_r_ with a difference of ca. 0.23 eV, but increase from 0.24 eV (AM1-DH+) to 0.28 eV (PM6-DH+) to 0.29 eV (RM1) in case of P_g_. We want to point out that the deviations in terms of RMSD values and excitation energies for the in vacuo optimized structures are expected to be significantly higher than in case of the protein-embedded chromophore, as the protein environment introduces additional constraints on the PCB geometry.

### 2.2. Spectrum Simulations Based on Sampling from Molecular Dynamics

**Figure 2:**
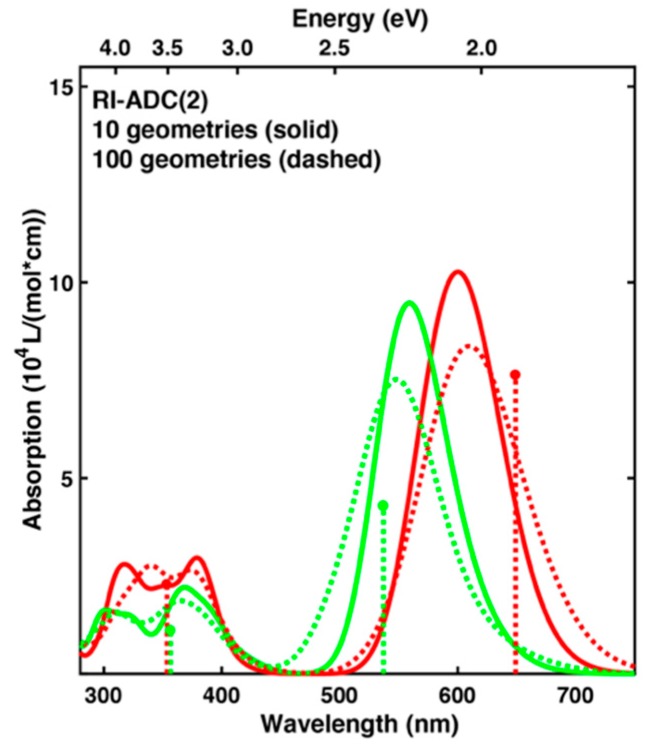
Absorption spectra for the P_r_ (red) and P_g_ (green) forms calculated with RI-ADC(2)/cc-pVDZ for QM66. The spectra are either based on 10 snapshots from a QM/MM MD trajectory taken every 100 ps (solid) or on 100 snapshots taken every 10 ps from the same trajectory (dotted). The sticks represent the positions and relative intensities of the two lowest energy absorption maxima for each form extracted from the measured spectra [14].

The QM/MM calculation of excitation energies used a QM region of at least 66 atoms (QM66), see Scheme 1. For efficient assessment of different methods, we decided to select a subset of 10 snapshots for each form. These leads to small changes in the overall spectra, but the qualitative features remain the same. A comparison of spectra based on RI-algebraic diagrammatic construction to second order (ADC(2)) for 10 or 100 snapshots is shown in Figure 2.

The discussion will mainly address the lowest energy absorption band, but for completeness the spectra simulated for all methods listed in Table 2 and Tables S7–S9 are given at the end of the Supplementary Materials in Figures S3–S6. In case of 10 snapshots, the lowest energy absorption band of both forms is narrower and more intense. Furthermore, the absorption maxima are shifted more towards each other. Nonetheless, a difference in absorption is 0.15 eV, see Table 2. Also the ratio of absolute absorption intensity of P_g_ divided by P_r_ remains close to 0.9 and is therefore hardly affected. Employing a larger cutoff of 24 Å for the point charges from the environment leads to a slightly smaller photoproduct tuning, but the absorption shifts are only around 0.01 eV for both forms and therefore the smaller cutoff of 12 Å appears to be sufficient. Employing a larger basis set including diffuse functions red shifts the absorption maxima of both forms to nearly the same extent, ca. 0.06 eV, leaving the photoproduct tuning virtually unchanged. The same holds for the ratio of absolute absorption intensities, where the larger basis set leads to a similar decrease for both forms. 

Strikingly, all tested wavefunction-based methods result in a similar photoproduct tuning of around 0.16 eV, even though the absorption is increasingly blue shifted for both forms starting with RI-CC2 (blue shift of around 0.15 eV relative to the RI-ADC(2) calculations with the same settings), time-dependent Hartree-Fock (TD-HF) (ca. 0.46 eV) to RI-coupled cluster singles (CCS) and configuration interaction singles (CIS) (ca. 0.70 eV). We note that the ratio of absolute absorption intensities is nearly the same for RI-ADC(2) and RI-CC2, but reduces to around 0.8 for the other three methods. This is contrary to the results obtained from TD-DFT, where the photoproduct tuning appears strongly dependent on the amount of exact exchange. 

A tuning similar to the one obtained via wavefunction-based approaches could only be achieved with the range-separated hybrid Coulomb-attenuating method (CAM)-B3LYP functional in case of TD-DFT, but the absorption maxima are significantly blue shifted. This is alleviated by employing functionals with smaller amount of exact exchange. The global hybrid functional B3LYP exhibits an absorption maximum of the P_g_ form close to the RI-ADC(2) calculations, whereas the absorption of the P_r_ form is significantly blue shifted leading to a relatively small photoproduct tuning of 0.08 eV. To better describe the absorption of the latter form, one could for example employ the GGA functional BLYP, but this functional exhibits a negligible tuning caused by the red-shifted absorption of the photoproduct. 

The use of the Tamm-Dancoff approximation (TDA) in combination with the B3LYP functional is also discouraging, as it blue shifts the absorption energies and in particular further reduces the already underestimated tuning found for full TD-DFT calculations with B3LYP. To conclude, we recommend the use of wavefunction-based methods to reliably estimate the difference in absorption between the two forms. In case of structures sampled from DFTB2+D/AMBER trajectories, RI-ADC(2) calculations result in absorption spectra that are the closest to the experimental counterparts. 

Semiempirical methods would allow more extensive excitation energy calculations for the spectrum simulation. Motivated by this, we have reported results from simplified TD-DFT (sTD-DFT) based on CAM-B3LYP ground state calculations and Zerner’s intermediate neglect of differenctial overlap for spectra (ZINDO/S) applied to 100 snapshots in the Supplementary Materials of Reference [12]. We found that ZINDO/S calculations lead to red-shifted absorption spectra and a slight underestimation of photoproduct tuning. sTD-DFT yields a slightly more pronounced photorproduct tuning than RI-ADC(2), but apart from that it is in good agreement with experiments, see Table S7. Regarding the changes in spectra, when employing 10 snapshots only and also when then employing a larger cutoff of 24 Å, we find a similar behavior as for RI-ADC(2). Furthermore, employing the simplified Tamm-Dancoff approximation (sTDA) instead of full sTD-DFT again blue shifts the lowest energy absorption band of both forms by around 0.14 eV and slightly reduces the extent of the photoproduct tuning. A more drastic change is found for the maximum values of absorption that have increased. Performing sTD-DFT based on DFT ground state calculations with ωB97 functional also leads to blue shifts in absorption spectra decreasing the agreement with the RI-ADC(2) results. To sum up the results, we recommend using sTD-DFT based on CAM-B3LYP ground state calculations for semiempirical excited state calculations, as the resulting simulated spectra are in good agreement with RI-ADC(2) reference results. The similarity is also confirmed for changes due to a reduced number of snaphots and an increased cutoff value. 

We have also probed the influence of the QM region size on the absorption spectra. For this purpose, we employed a larger QM region consisting of the complete PCB chromophore, the bound cysteine sidechain and the sidechains of HIS-529 and ASP-498, which are close to the conjugated system of PCB. This results in a QM region containing 106 atoms (QM106). Further details regarding this QM region can be found in our previous publication [12]. In case of the RI-ADC(2) calculations for QM106, we have employed a reduced virtual space (RVS, [84,85,86,87]) by discarding all virtual molecular orbitals with energies above 50 eV. As can be seen by comparison of the values reported in Table S8, RVS leads to a small blue shift for the lowest energy absorption maximum of both forms by ca. 0.03 eV, however, the difference in absorption remains essentially unchanged. However, this shift should be taken into account when comparing the results from QM106 to the ones from QM66. In case of 100 snapshots, the lowest energy absorption maxima are nonetheless shifted to lower energies and the shift is more pronounced for P_r_ than for P_g_ resulting in an increased difference in absorption of 0.26 eV. Similar shifts are also found when comparing 10 snapshots for both QM regions. In addition, also the ratio in peak heights goes down from ca. 0.90 to 0.80, closer to the experimental ratio of 0.56. So the deviations for the positions of the absorption maxima are more symmetric and the peak heights are also more similar to the experiments. In case of TD-DFT calculations with B3LYP, the absorption maxima of both forms are shifted by nearly the same amount of ca. 0.06 eV, hence the difference in absorption is again underestimated. For the ratio in absorption height, we observe a similar decrease by around 0.1 as for RI-ADC(2). The shifts towards lower energies for ZINDO/S are less prominent, but as in case of the RI-ADC(2) calculations, they are more pronounced for P_r_ than for P_g_ leading to larger differences between the absorption maxima of both forms. Also the ratio in peak heights decreases by around 0.05. This holds also for sTD-DFT calculations, but the shifts in positions are less systematic, whereas the difference in absorption remains around the same in the comparison of the two QM regions. 

For the ab initio multi-reference calculations it was technically not feasible to include all π and π* orbitals as well as lone pairs in the active space, which would result in 24 electrons in 22 orbitals. Instead we employed an active space of 20 electrons in 13 orbitals, as RI-CC2 calculations for the PCB chromophore in protein conformation of both forms indicated the involvement of up to 10 occupied and 3 unoccupied orbitals for all states up to 4.2 eV. Based on this active space selection, we calculated excited states with both the partially- (PC) and strongly-contracted (SC) N-electron valence state perturbation theory to second order (NEVPT2) scheme, see Table S9. The position of the absorption maximum for the P_g_ form for SC-NEVPT2 is in close agreement with the experimental value, whereas PC-NEVPT2 leads to a slight red shift. However, the absorption of P_r_ is blue shifted by 0.20 eV and 0.32 eV for PC- and SC-NEVPT2, respectively. This leads to a photoproduct tuning of only 0.07 eV (PC-NEVPT2) and 0.08 eV (SC-NEVPT2). Also the absorption intensities are now interchanged, i.e., the P_g_ absorption maximum is slightly higher than the one of P_r_. In particular the blue shifted absorption for P_r_ might indicate that a larger active space is required to better describe the more conjugated structures of this form. 

Calculations with a larger active space are possible with semiempirical multi-reference methods. We employed multi-reference configuration interaction (MRCI) based on ground state calculations with the orthogonalization-corrected method 2 (OM2). Including the 20 highest occupied and 20 lowest unoccupied orbitals resulting in an active space of 40 electrons in 40 orbitals (40/40) yields an photoproduct tuning of 0.17 eV, where again the lowest energy absorption maximum of P_g_ matches its experimental counterpart at 2.31 eV and the P_r_ absorption maximum is blue-shifted by 0.23 eV. Selecting only 10 occupied and 10 unoccupied orbitals that possess a certain amount of π orbital character leads to further blue shifts of both absorption bands but leaves the photoproduct tuning virtually unchanged. However, this smaller active space allows to include triples, which decrease the energy for the lowest energy absorption maximum of both forms by nearly 0.08 eV. Overall, the employed semiempirical multi-reference calculations always yield a photoproduct tuning that is slightly higher than for the wavefunction-based calculations and somewhat lower than for the corresponding sTD-DFT calculations. However, as for the ab initio multi-reference calculations there is a tendency to overestimate the excitation energies in particular for P_r_. Furthermore, absorption intensities are found to be around the same for both forms within each of the three employed MRCI settings, placing the OM2-MRCI results between the ones from NEVPT2 and single-reference methods in this regard. 

### 2.3. Spectrum Simulations Based on QM/MM Geometry Optimizations

In this section we have used geometries from MD simulations, reported in Ref. [12], as a starting point for QM/MM geometry optimization at the RI-BLYP+D3/AMBER level of theory using the ChemShell program. For the geometry optimization we have employed three different approaches: (i) QM106 in cartesian coordinates, (ii) QM66 in cartesian as well as (iii) in hybrid delocalized coordinates (HDLC). After optimization, the structures are aligned on the basis of 42 atoms as previously discussed for the gas phase comparison. The resulting RMSD values between the three different approaches are below 0.10 Å for both forms, see Table S10. In addition, the QM66 optimized structures are even closer to each other with RMSD values of around 0.03 Å. This is also reflected in the spectra based on sTD-DFT with QM106 shown in Figure S1. This figure shows that the spectra from QM66 optimizations are close to each other for each form and also QM106 exhibits only small differences to the less expensive approaches. We therefore recommend using QM66 with HDLC for full structure optimizations, when one wants to treat the QM part with ab initio electronic structure methods. In our case this leads to convergence in around 5000 steps for systems consisting of ca. 40,000 atoms. 

**Figure 3:**
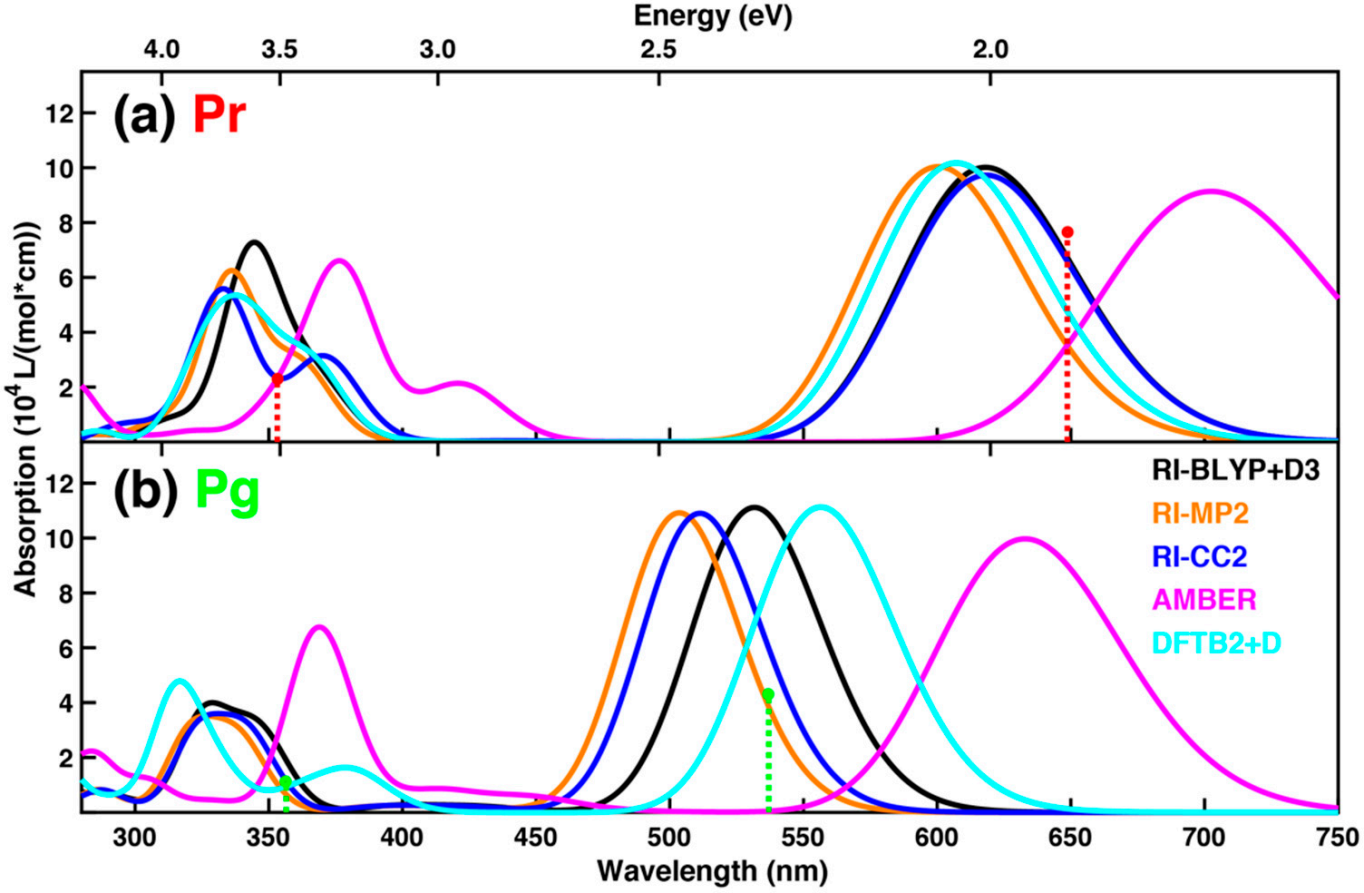
QM/MM absorption spectra for structures optimized at different levels of theory with QM66 for (**a**) the P_r_ and (**b**) the P_g_ form. The method for the treatment of the QM atoms is stated in the legend, while AMBER denotes pure force field calculations. Excitation energies are obtained using sTD-DFT calculations for QM106. The sticks represent the positions and relative intensities of the two lowest energy absorption maxima for each form extracted from the measured spectra [14].

For further improvement of the level of theory with the QM66 scheme, we have employed RI-Møller-Plesset perturbation theory to second order (MP2) and RI-CC2 in combination with the AMBER force field starting from the RI-BLYP+D3/AMBER optimized structures. These optimizations converge in around 100 steps and the RMSD values for alignment of structures optimized with these ab initio methods is below 0.10 Å, see Table 3. Therefore, the pure GGA functional BLYP with additional dispersion correction appears to be a reliable method for structure description of the PCB chromophore in the protein. In contrast to this, larger differences are found, when the structures are only optimized with the AMBER force field: The RMSD value is then around 0.16 Å for P_r_ and 0.29 Å for P_g_ relative to RI-CC2. This is also reflected in pronounced red shifts of the lowest energy absorption band for each form, see Figure 3. In case of optimizations with DFTB2+D, we have employed several approaches to optimize the structures as shown in Table S12 and Figure S2. The differences between them are relatively small and therefore, in the main text we just show the results of the initial structure optimization with 100,000 steps of steepest descent starting from the last snapshot of the production run, which was also the starting point for the BLYP+D3/AMBER and pure AMBER force field optimizations. DFTB2+D exhibits RMSD values of 0.12 Å (P_r_) and 0.19 Å(P_g_) placing it in between the pure force field treatment and the ab initio methods for QM/AMBER calculations, see Table 3. In addition, the spectra resulting from the DFTB2+D optimized structures are closer to the ones from ab initio QM/MM optimizations than from pure force field optimizations, see Figure 3. However, the absorption maximum for the P_g_ form is red-shifted relative to the ab initio methods resulting in a photoproduct tuning that is similar to the ones based on force field optimized structures and somewhat smaller than for QM/AMBER optimized ones. Nonetheless, the accuracy of DFTB2+D is reasonably close to the ab initio methods, but it has the advantage of being ca. 2 orders of magnitude faster than RI-BLYP+D3/def2-SV(P), thus allowing more extended MD for sampling. Due to the relatively poor description of the geometry and red-shifted absorption for the protein-embedded PCB chromophore by force field optimization, care should be taken when sampling from classical MD without any further refinements. A similar conclusion has been reported by González and coworkers in a QM/MM study of the temoporfin absorption spectrum [88].

### 2.4. Comparison of Absorption Spectra: Sampling versus Optimized Structures

**Figure 4:**
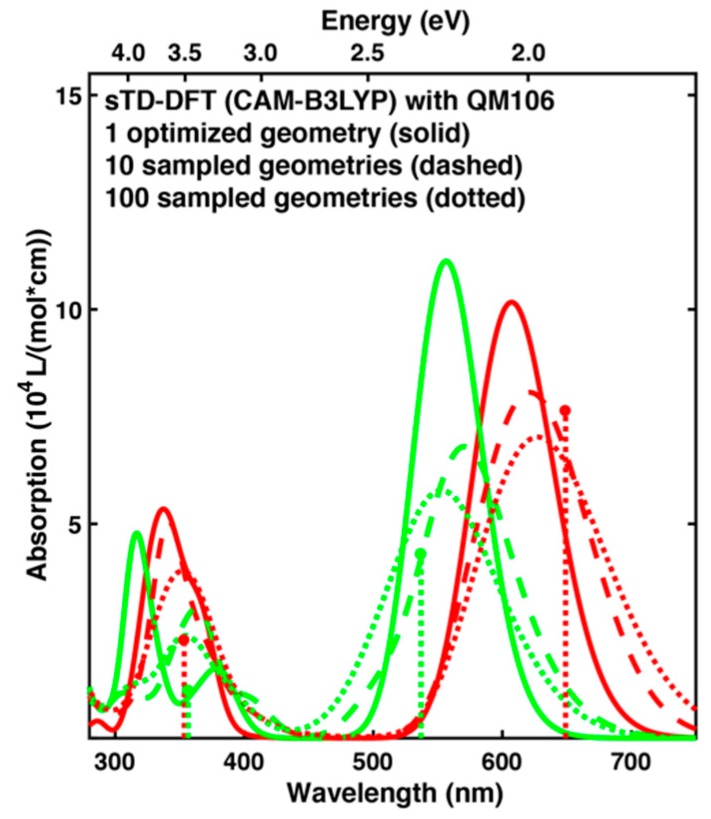
Absorption spectra for the P_r_ (red) and P_g_ (green) forms calculated with sTD-DFT for QM106. The spectra are either based on one singly optimized structure (solid), 10 snapshots from a QM/MM MD trajectory taken every 100 ps (dashed) or 100 snapshots taken every 10 ps from the same trajectory (dotted). The sticks represent the positions and relative intensities of the two lowest energy absorption maxima from experiment [14].

Using the DFTB2+D/AMBER method one can compare calculated spectra based on optimized and sampled geometries, see Figure 4. For the lowest energy absorption band, the positions of the maximum change slightly, but there is hardly any systematic trend: For the P_r_ form the maximum is shifting towards longer wavelengths when going from optimized structure, to 10 and 100 snapshots. However, for P_g_ the maximum first red shifts, but then moves back close to its original position. In contrast to this, the more structures are considered, the broader and less intense this first absorption band becomes for both forms.

This is also observed for the second absorption band, but in addition we find that the distinct peaks found in case of optimized structures are washed out, so that these absorption bands becomes less structured. This improves the agreement with experiments, where the second absorption band of both forms also appears broad without much fine structure [13,14]. Overall, the photoproduct tuning might be already described with one or few selected structures, whereas sampling is necessary to describe the shape of the absorption bands and their broadening due to temperature effects, which was also stated in a recent study investigating chromophores in solution with QM/MM [89]. We note that with the approaches employed by us, it is not possible to describe vibronic effects as reviewed recently [90,91]. 

## 3. Discussion

In this contribution, we have investigated essential parts of a computational protocol to model the absorption of both P_r_ and P_g_ forms from the same CBCR protein Slr1393g3. First, we have assessed the description of the in vacuo optimized PCB geometries using semiempirical methods by comparing them with structures optimized with the ab initio RI-CC2 method. The best performing method, DFTB2+D, was used to simulate QM/MM MD trajectories with a simulation time of 1 ns and snapshots were extracted for excited state calculations with electrostatic embedding. For this purpose, we recommend the use of wavefunction-based methods, as these methods result in similar photoproduct tunings. In particular RI-ADC(2) has proven to work well with the structures that were generated via DFTB2+D/AMBER MD, resulting in positions of the lowest energy absorption maxima close to the experimental counterparts. 

Furthermore, we stress that caution should be exercised, when TD-DFT is employed: A tuning similar to the one from the aforementioned methods is only obtained with a range-separated hybrid functional, but then excitation energies are overestimated. On the other hand, BLYP as a prototypical GGA functional leads to a negligible tuning, but it is the best functional to calculate the absorption spectrum for the P_r_ form in agreement with previous benchmark studies [44,51]. Furthermore the root mean square electron hole separation, which can be interpreted as exciton size [92], for the first excited state S_1_ obtained as an average over 10 snapshots for P_r_ increases from CAM-B3LYP (5.06 Å) to B3LYP (5.90 Å) to BLYP (6.16 Å), whereas the corresponding result for RI-ADC(2) is 5.11 Å, see Table S15. So the size of the exciton is similar for CAM-B3LYP and RI-ADC(2), but this functional results in an overestimation of excitation energy, as can be seen by the simulated spectra as well as by the averaged energies of the first excited state. In contrast to this, the first excitation energies and the corresponding lowest energy absorption band from BLYP are closer to the RI-ADC(2) results, but it is on the expense of the correct exciton description. Similar conclusions were also reached in a study by Mewes et al. investigating the absorption of Magnesium(II)porphyrin [93]. This deficiency in the description of the exciton properties is also found for the P_g_ form and might be the underlying reason for the failure of the employed GGA functional to yield a reliable photoproduct tuning. It might be possible to improve the quality of the TD-DFT results by employing optimally tuned range-separated hybrid functionals [94]. 

However, as an alternative for now, we recommend using sTD-DFT based on CAM-B3LYP ground state calculations. Its superior performance might be traced back to the fact that the parameterization of the related sTDA method was done for range-separated hybrid functionals on the basis of SCS-CC2 calculations [95]. The fit set included the closed tetrapyrrole porphyrine and the CAM-B3LYP functional performed the best for the description of valence excited states like ππ* excitations [95], which are the origin of the first two absorption bands. Furthermore, also ZINDO/S, which has been successfully employed to study related tetrapyrroles [96,97], performs rather well, even though the absorption bands are somewhat red-shifted and the tuning is slightly underestimated. An advantage of employing semiempirical calculations is their efficiency. They were run in serial on nodes with one Intel Xeon processor E3-1230. For QM66, a typical ZINDO/S calculation takes about 10 s, the DFT ground state calculation for sTD-DFT takes about 1 h, but the actual excited state calculation requires 10 s. For QM106, ZINDO/S requires around 40 s, the CAM-B3LYP ground state calculation approximately 4 h and the sTD-DFT excited state calculation between 1 and 2 min. In contrast, each RI-ADC(2) calculation was run in parallel on one node with two Intel Xeon E5-2670 processors using 16 cores. Nonetheless, the wall times for QM66 were around 7–8 h and 4–5 days for the larger QM106. For all calculations, no significant influence of the employed cutoff radius for the point charges on the computing time was observed. 

The comparison of optimized structures to the RI-CC2/AMBER reference geometries shows that DFTB2+D/AMBER improves the description relative to a purely classical treatment. As shown in this study, a reasonable photoproduct tuning can be obtained by sampling structures from DFTB2+D/ AMBER MD, but based on the results for the optimized structures, in particular the PCB geometry of the P_g_ form appears to be challenging. To improve the quality of the sampled structures, one may either resort to more recent semiempirical methods based on tight binding, like extended Tight Binding (xTB) [98,99] or DFTB3 with Grimme′s dispersion correction D3 [83]. In addition, xTB could also be employed to speed up the sTD-DFT calculations [100]. As an alternative approach for sampling, one may use structures sampled from DFTB2+D/AMBER MD as starting points for more accurate QM/MM MD. In case of the latter, RI-BLYP+D3/def2-SV(P) appears promising as the structures and excitation energies are close to the computationally more demanding RI-MP2 and RI-CC2 calculations. We expect that this approach will increase the simulated photoproduct tuning, but for computational reasons the corresponding trajectories might only be computed for several ps. In addition, further deviations of the simulated absorption spectra from the experimental ones might be caused by the setup of the system. For example, this study was performed with a doubly protonated HIS-529, similar to previous studies on the related red/green CBCR AnPixJ, in which the analogue histidine was also assumed to be in this protonation state [16,17]. However, simulations with a singly protonated HIS-529 lead to absorption spectra in better agreement with experiments, see the Supplementary Materials of reference [12]. Furthermore, sampling of different PCB conformations might be required to improve the agreement with experiments, as Scarbath-Evers et al. have shown that in case of the P_r_ form of the closely related red/green CBCR AnPixJg2 two substates are found in classical MD simulations [17]. These substates differ in the orientation of the D ring, however, switching between both forms happens on a time scale of hundreds of nanoseconds. 

Overall, we expect that the protocol outlined in this benchmark study to investigate the spectral tuning in a prototypical red/green CBCR is also applicable to further proteins of the CBCR family but also to a broad range of canonical phytochromes. Therefore, it can serve as a reference for future studies on spectral tuning in these types of photoreceptor proteins.

## 4. Materials and Methods 

### 4.1. Chromophore Structure Optimizations with Semiempirical Methods

The chromophore geometries were taken from the crystal structures of the in vivo assembled forms, i.e., PDB codes 5DFX for P_r_ and 5M82 for P_g_ [24]. Missing hydrogen atoms were added via GaussView [101] and side chains as well as methyl groups were replaced with hydrogen atoms with the same program resulting in truncated chromophores with 42 atoms for both forms. These structures were optimized with the SQM module of the AMBER software suite [59] until the default convergence criteria were reached and with all implemented semiempirical quantum chemical methods that are parameterized for the elements H, C, N, O, and S. For the same structures, RI-CC2/cc-pVDZ optimizations [60,61,62,63] were also performed with Turbomole 7.0 [64,65] to provide a benchmark for comparison of the differently optimized structures. For this purpose, the optimized geometries were aligned via PyMOL [102] including all atoms. Furthermore, excited state calculations were also performed with RI-CC2/cc-pVDZ [103,104] for the differently optimized structures to assess their influence on excitation energies and oscillator strengths. The RI-CC2 calculations employed frozen core orbitals and in case of the excited state calculations, a threshold of 10^−8^ in atomic units for the SCF calculations was used. Otherwise, the default values were taken. 

### 4.2. Spectrum Simulations Based on Sampling from Molecular Dynamics

The structures were taken from the previously published trajectory [12], which was simulated employing the hybrid QM/MM methodology [105]. In the following, the employed computational approach is described in a nutshell and full details can be found in the Supplementary Materials of [12]. Hydrogen atoms were added via tleap from the AMBER software package [59] to the crystal structures of the in vivo assembled forms, i.e., PDB codes 5DFX for P_r_ and 5M82 for P_g_ [24]. The structures were solvated with TIP3P water molecules [106] in rectangular boxes with a distance of at least 15 Å between any atom of the protein and the boundary. Standard protonation states were employed with the exception of HIS-529, which is assumed to be doubly protonated. For the non-bonded interactions, a cutoff of 12 Å was chosen. Initial optimizations consisted of three stages:

(i) Optimization via AMBER ff14SB force field [107] of the environment employing harmonic restraints of 500 kcal/(mol Å^2^) on the atoms of the protein with respect to their crystallographic positions.
(ii) Optimization via AMBER of the whole system with restraints on the atoms of the modified residue, i.e., on the PCB chromophore and CYS-528, which binds PCB.
(iii) DFTB2+D/AMBER [57,58,77,78,82,108] optimizations without any restraints.

Also the subsequent molecular dynamics (MD) simulations consisted of three stages:

(i) Thermalization with classical MD with stepwise increase of the temperature from 0 to 300 K within 1 ns employing restraints of 10 kcal/(mol Å^2^) on the modified residue to keep the geometry close to the DFTB2+D/AMBER optimized one.
(ii) Equilibration via classical MD for 100 ns at 300 K to allow backbone relaxation employing the same restraints as before.
(iii) Production run with DFTB2+D/AMBER for 1 ns at 300 K without any restraints.

Originally, snapshots were taken every 10 ps leading to 100 snapshots for spectrum simulation. In this contribution, we have decided to resort to 10 snapshots taken every 100 ps, as the qualitative features of the spectra are also present then and the reduced number of geometries allows a broader assessment of methods. For the excited state calculations and if not stated otherwise, the protein environment was taken into account as point charges with a cutoff distance of 12 Å to any of the atoms in the QM region. Excitation energies and oscillator strengths obtained from these calculations were finally broadened with Gaussian functions employing a full width at half maximum of 0.25 eV and the spectra shown in case of MD are averages of at least 10 snapshots. 

Regarding the ab initio single-reference methods, the cc-pVDZ basis set [60] was employed and post-Hartree-Fock calculations (RI-CCS, RI-ADC(2), and RI-CC2 [61,103,104,109,110,111]) were performed with Turbomole 7.0 [64,65], whereas for CIS, TD-DFT (BLYP, B3LYP, CAM-B3LYP, and B3LYP with Tamm-Dancoff approximation [112,113,114,115,116]), and TD-HF calculations [117,118] the Gaussian software package [119] was employed. In case of the smaller QM region consisting of the chromophore and 66 atoms in total (QM66), 10 excited states were determined, whereas for the larger QM region varying numbers of excited states were calculated: 30 for B3LYP, 15 for RI-ADC(2) with a reduced virtual space (RVS) [84,85,86,87] employing a cutoff of 50 eV for orbital energies and 10 for RI-ADC(2) calculations with a full virtual space (FVS). This choice is based on the finding that 15 excited states are sufficient for QM106 to model the first and second absorption band. Furthermore, for computational reasons we decided to calculate even fewer states for the calculations with a full virtual space, which is nonetheless sufficient to probe the influence of discarding virtual orbitals in the excited state calculations on the lowest energy absorption band. Details of the further settings for the Turbomole calculations are given in Section 4.1 and for Gaussian 09 the default ones were taken. Finally, the root mean square electron hole separation for the calculations described in this paragraph were determined with TheoDORE [92,120,121].

Orca version 4.0.1.2 [122,123] was employed for the semiempirical ZINDO/S [96,124] and sTD-DFT/sTDA [95,125,126] calculations. Independent of the QM region, 30 excited states were calculated with the former method and all states up to 10 eV with the latter. Furthermore, the ground state calculations for sTD-DFT/sTDA were performed mainly with the range-separated CAM-B3LYP hybrid functional [115], but also with ωB97 [127]. The def2-SV(P) basis set [128] and tight SCF convergence criteria were employed for these calculations. 

Multi-reference calculations were only performed for QM66. The ab initio ones with NEVPT2 [129,130,131] employed MOLPRO, version 2015.1 [132,133]. They are based on state-averaged complete active space self-consistent field calculations of the first six electronic states, for which also excitation energies and oscillator strengths from ground to excited states were determined. Based on an inspection of the low-lying excited states from RI-CC2 calculations for the truncated PCB chromophore in protein conformation, it was decided to include 10 occupied molecular orbitals (MO) and 3 unoccupied MOs. The semiempirical multi-reference calculations were performed with the MNDO program [134] and are based on OM2 ground state calculations [135] with an open-shell singlet ansatz for the wavefunction. The subsequent MRCI calculations [136] employed three reference states and an active space consisting of the ten highest occupied and ten lowest unoccupied MOs, therefore following the approach described in Ref. [137] and employing the algorithm described there for π orbital selection. This is motivated by the finding that the low-lying bright transitions are dominated by ππ* excitations. For this setup, we have also assessed the influence of triples on the simulated absorption spectra. In addition, MRCI calculations using six reference states and an active space consisting of the 20 highest occupied and 20 lowest unoccupied MOs were also performed.

### 4.3. Spectrum Simulations Based on QM/MM Geometry Optimizations

The classical AMBER, DFTB2+D/AMBER and RI-BLYP+D3/AMBER optimizations started from the final structures of the 1 ns trajectories and in case of the latter two approaches employed QM66, if not stated otherwise. The optimizations were performed with the DL-FIND module [138] in ChemShell [139,140,141] with an exception being the DFTB2+D/AMBER ones [57,58,77,78,82,108], which were realized directly with the AMBER software package [59] employing 100,000 steps of steepest descent. In all optimizations, no atoms were frozen and no restraints were applied. For the DFT-based optimizations, the Orca-ChemShell interface was employed and the GGA functional BLYP [112,113] was utilized together with the def2-SV(P) basis set [128], Grimme′s dispersion correction D3 [83] with Becke-Johnson damping [142], and the resolution-of-identity approximation (RI) with corresponding density fitting basis set [143]. In case of the RI-MP2/AMBER and RI-CC2/AMBER [63,144,145,146] optimizations with QM66 and cc-pVDZ basis set, ChemShell was interfaced with Turbomole and owing to the relatively high computational demands, the starting structures were taken from the RI-BLYP+D3/AMBER optimizations. The optimized structures were then used for excited state calculations employing sTD-DFT as described before for QM106, owing to its good agreement with the more demanding ab initio RI-ADC(2) calculations and relatively low computational costs. Furthermore, the optimized structures were also truncated to 42 atoms and aligned as described in Section 4.1.

## Figures and Tables

**Table 1:** Root mean square deviations of geometries in Å. Comparisons are given for methods in the corresponding column and row for P_r_ (first value) and P_g_ (second value, shown in parenthesis).

P_r_ (P_g_)	RM1	AM1-DH+	DFTB2+D	RI-CC2
**PM6-DH+**	0.18 (0.35)	0.35 (0.25)	0.29 (0.96)	0.76 (1.10)
**RM1**	-	0.32 (0.23)	0.31 (0.90)	0.65 (1.02)
**AM1-DH+**		-	0.39 (0.81)	0.67 (0.96)
**DFTB2+D**			-	0.58 (0.23)

**Table 2:** Lowest energy absorption maxima of P_r_ and P_g_. Wavelengths (λ), energies (E_max_), energy differences in parenthesis and absolute absorption (ε_Pr_ and ε_Pg_) as well as photoproduct tuning (ΔE_max_) and ratio of absorption intensities (ε_Pg_/ε_Pr_) are tabulated. The QM region consisted of 66 atoms and if not stated otherwise, the results are based on 10 snapshots taken every 100 ps. The cc-pVDZ basis set was utilized and a cutoff of 12 Å to any of the QM atoms was employed to take the environment as point charges into account.

Method		P_r_			P_g_		Comparison	
	λ (nm)	E_max_ (eV)	ε_Pr_^3^	λ (nm)	E_max_ (eV)	ε_Pg_^3^	ΔE_max_ (eV)	ε_Pg_/ε_Pr_
**Exp.^1^**	649	1.91	-	536	2.31	-	0.40	0.562
**RI-ADC(2)**								
100 geom.^2^	609	2.04 (+0.13)	8.37	548	2.26 (−0.05)	7.52	0.23	0.898
10 geom. ^2^	600	2.07 (+0.16)	10.27	559	2.22 (−0.10)	9.47	0.15	0.922
24 Å cutoff	596	2.08 (+0.17)	9.32	561	2.21 (−0.10)	9.13	0.13	0.979
cc-aug-pVDZ^2^	616	2.01 (+0.10)	9.83	575	2.16 (−0.16)	9.03	0.14	0.918
**WF-based**								
RI-CC2	560	2.21 (+0.30)	12.77	524	2.37 (+0.05)	11.56	0.15	0.905
RI-CCS	448	2.77 (+0.86)	14.24	423	2.93 (+0.62)	11.39	0.16	0.800
CIS	448	2.77 (+0.86)	14.45	423	2.93 (+0.62)	11.47	0.17	0.794
TD-HF	493	2.52 (+0.61)	12.59	461	2.69 (+0.37)	9.99	0.17	0.793
**DFT-based**								
CAM-B3LYP	538	2.31 (+0.40)	11.98	504	2.46 (+0.15)	10.70	0.15	0.893
B3LYP	569	2.18 (+0.27)	10.98	548	2.26 (−0.05)	8.68	0.08	0.790
B3LYP (TDA)	500	2.48 (+0.57)	17.38	497	2.49 (+0.18)	11.48	0.01	0.661
BLYP	609	2.03 (+0.12)	9.00	609	2.04 (−0.28)	5.68	0.00	0.631

^1^ Taken from the spectra in Reference [14]. ^2^ Values as reported in our previous publication [12]. ^3^ In units of 10^4^ L/(mol cm).

**Table 3:** Root mean square deviations of geometries in Å. Comparisons are given for methods in the corresponding column and row for P_r_ (first value) and P_g_ (second value, shown in parenthesis). For alignment, the geometries were reduced to 42 atoms as in case of the optimizations in vacuo. They were obtained from QM/AMBER optimized structures with QM66, while AMBER denotes pure force field calculations.

P_r_ (P_g_)	DFTB2+D	RI-BLYP+D3	RI-MP2	RI-CC2
**AMBER**	0.14 (0.24)	0.16 (0.27)	0.16 (0.30)	0.16 (0.29)
**DFTB2+D**	-	0.12 (0.15)	0.12 (0.19)	0.12 (0.19)
**RI-BLYP+D3**		-	0.06 (0.07)	0.05 (0.07)
**RI-MP2**			-	0.01 (0.01)

## Supplementary Materials

The following are available online, Tables S1–S4, S10 and S12: Root mean square deviations of geometries in Å, Tables S5 and S6: Excitation energies and oscillator strengths for in vacuo optimized structures, Tables S7–S9, S11, S13 and S14: Lowest energy absorption maxima of both forms, Table S15: Results from wave function analysis based on sampled structures, Figures S1 and S2: Absorption spectra from optimized structures, Figures S3–S6: Absorption spectra from sampling.
