# Supplementary material for: QM/MM Benchmarking of Cyanobacteriochrome Slr1393g3 Absorption Spectra
Source: Molecules. 2019 May 3;24(9):1720. doi: 10.3390/molecules24091720 (PMC6540152; doi:10.3390/molecules24091720)
Supplement: Supplementary file 1 [file molecules-24-01720-s001.pdf]

molecules

*Supplementary Materials*

*Article*

# QM/MM Benchmarking of Cyanobacteriochrome Slr1393g3 Absorption Spectra

**Christian Wiebeler † and Igor Schapiro ***

Fritz Haber Center for Molecular Dynamics Research, Institute of Chemistry, The Hebrew University of Jerusalem, Jerusalem 91904, Israel; chwie@mail.upb.de
* Correspondence: Igor.Schapiro@mail.huji.ac.il
† Current address: Physics Department, Universität Paderborn, 33098 Paderborn, Germany

**Table 1.** Root mean square deviations of geometries in Å. Comparisons are given for methods in the corresponding column and row for $P_r$ (first value) and $P_g$ (second value, shown in parenthesis).

| $P_r$ ($P_g$) | PM3 | PM3-PDDG | RI-CC2 |
|---|---|---|---|
| **MNDO** | 0.54 (0.67) | 0.53 (0.59) | 0.98 (1.27) |
| **PM3** | - | 0.28 (0.54) | 0.99 (1.26) |
| **PM3-PDDG** | | - | 1.15 (1.60) |

**Table S2.** Root mean square deviations of geometries in Å. Comparisons are given for methods in the corresponding column and row for $P_r$ (first value) and $P_g$ (second value, shown in parenthesis).

| $P_r$ ($P_g$) | PM6-D | PM6-DH+ | RI-CC2 |
|---|---|---|---|
| **PM6** | 0.06 (0.15) | 0.11 (0.27) | 0.87 (1.34) |
| **PM6-D** | - | 0.06 (0.14) | 0.82 (1.22) |
| **PM6-DH+** | | - | 0.76 (1.10) |

**Table S3.** Root mean square deviations of geometries in Å. Comparisons are given for methods in the corresponding column and row for $P_r$ (first value) and $P_g$ (second value, shown in parenthesis).

| $P_r$ ($P_g$) | AM1/d | AM1-D | AM1-DH+ | RM1 | RI-CC2 |
|---|---|---|---|---|---|
| **AM1** | 0.27 (0.37) | 0.19 (0.27) | 0.24 (0.28) | 0.35 (0.40) | 0.85 (1.17) |
| **AM1/d** | - | 0.16 (0.25) | 0.18 (0.24) | 0.37 (0.12) | 0.73 (1.10) |
| **AM1-D** | | - | 0.13 (0.13) | 0.31 (0.21) | 0.68 (0.96) |
| **AM1-DH+** | | | - | 0.32 (0.23) | 0.67 (0.96) |
| **RM1** | | | | - | 0.65 (1.02) |

**Table S4.** Root mean square deviations of geometries in Å. Comparisons are given for methods in the corresponding column and row for $P_r$ (first value) and $P_g$ (second value, shown in parenthesis).

| $P_r$ ($P_g$) | DFTB3 | DFTB2+D | RI-CC2 |
|---|---|---|---|
| **DFTB2** | 0.12 (0.13) | 0.22 (0.80) | 0.79 (0.94) |
| **DFTB3** | - | 0.33 (0.92) | 0.89 (1.06) |
| **DFTB2+D** | | - | 0.58 (0.23) |

**Table S5.** Excitation energies (E, in eV) and oscillator strengths (f) for the first 10 excited singlet states for $P_r$ from RI-CC2/cc-pVDZ calculations based on structures optimized *in vacuo* with the method given as headline of the two columns.

| $P_r$ | RI-CC2 | | DFTB2+D | | DFTB2 | | DFTB3 | | AM1-DH+ | | PM6-DH+ | | RM1 | |
|---|---|---|---|---|---|---|---|---|---|---|---|---|---|---|
| | E | f | E | f | E | f | E | f | E | f | E | f | E | f |
| $S_1$ | 2.01 | 0.825 | 2.16 | 1.154 | 2.18 | 1.261 | 2.20 | 1.319 | 2.23 | 1.118 | 2.24 | 1.199 | 2.25 | 1.131 |
| $S_2$ | 3.19 | 0.228 | 3.35 | 0.237 | 3.39 | 0.230 | 3.40 | 0.000 | 3.37 | 0.271 | 3.35 | 0.300 | 3.33 | 0.299 |
| $S_3$ | 3.35 | 0.000 | 3.42 | 0.000 | 3.43 | 0.000 | 3.40 | 0.237 | 3.48 | 0.000 | 3.73 | 0.000 | 3.57 | 0.001 |
| $S_4$ | 3.78 | 0.410 | 3.85 | 0.363 | 3.85 | 0.361 | 3.88 | 0.352 | 3.99 | 0.213 | 3.92 | 0.121 | 3.98 | 0.054 |
| $S_5$ | 4.04 | 0.152 | 4.11 | 0.103 | 4.14 | 0.109 | 4.16 | 0.114 | 4.06 | 0.090 | 3.99 | 0.124 | 3.99 | 0.170 |
| $S_6$ | 4.08 | 0.006 | 4.28 | 0.045 | 4.30 | 0.038 | 4.30 | 0.037 | 4.24 | 0.089 | 4.24 | 0.097 | 4.23 | 0.095 |
| $S_7$ | 4.21 | 0.051 | 4.34 | 0.060 | 4.35 | 0.038 | 4.37 | 0.067 | 4.34 | 0.059 | 4.36 | 0.051 | 4.39 | 0.037 |
| $S_8$ | 4.40 | 0.295 | 4.56 | 0.156 | 4.38 | 0.034 | 4.43 | 0.008 | 4.49 | 0.000 | 4.70 | 0.243 | 4.58 | 0.003 |
| $S_9$ | 4.48 | 0.003 | 4.63 | 0.022 | 4.63 | 0.049 | 4.55 | 0.001 | 4.64 | 0.233 | 4.71 | 0.015 | 4.70 | 0.209 |
| $S_{10}$ | 4.71 | 0.150 | 4.65 | 0.110 | 4.63 | 0.222 | 4.65 | 0.266 | 4.91 | 0.097 | 4.94 | 0.090 | 4.99 | 0.126 |

**Table S6.** Excitation energies (E, in eV) and oscillator strengths (f) for the first 10 excited singlet states for $P_g$ from RI-CC2/cc-pVDZ calculations based on structures optimized *in vacuo* with the method given as headline of the two columns.

| $P_g$ | RI-CC2 | | DFTB2+D | | DFTB2 | | DFTB3 | | AM1-DH+ | | PM6-DH+ | | RM1 | |
|---|---|---|---|---|---|---|---|---|---|---|---|---|---|---|
| | E | f | E | f | E | f | E | f | E | f | E | f | E | f |
| $S_1$ | 2.00 | 0.747 | 2.06 | 0.828 | 2.17 | 1.211 | 2.19 | 1.271 | 2.24 | 1.061 | 2.28 | 1.125 | 2.29 | 0.994 |
| $S_2$ | 3.20 | 0.252 | 3.27 | 0.262 | 3.38 | 0.000 | 3.35 | 0.000 | 3.37 | 0.355 | 3.35 | 0.386 | 3.31 | 0.352 |
| $S_3$ | 3.24 | 0.001 | 3.31 | 0.001 | 3.41 | 0.219 | 3.43 | 0.250 | 3.44 | 0.011 | 3.71 | 0.000 | 3.58 | 0.001 |
| $S_4$ | 3.61 | 0.491 | 3.61 | 0.449 | 3.61 | 0.468 | 3.66 | 0.453 | 3.90 | 0.271 | 3.87 | 0.164 | 3.98 | 0.069 |
| $S_5$ | 4.02 | 0.014 | 4.06 | 0.008 | 4.19 | 0.010 | 4.21 | 0.012 | 4.06 | 0.025 | 3.98 | 0.029 | 4.01 | 0.191 |
| $S_6$ | 4.07 | 0.027 | 4.19 | 0.016 | 4.31 | 0.015 | 4.32 | 0.015 | 4.26 | 0.045 | 4.28 | 0.045 | 4.26 | 0.058 |
| $S_7$ | 4.21 | 0.068 | 4.28 | 0.056 | 4.35 | 0.015 | 4.38 | 0.028 | 4.36 | 0.067 | 4.36 | 0.082 | 4.38 | 0.064 |
| $S_8$ | 4.47 | 0.415 | 4.57 | 0.424 | 4.39 | 0.061 | 4.40 | 0.049 | 4.50 | 0.002 | 4.72 | 0.004 | 4.58 | 0.003 |
| $S_9$ | 4.52 | 0.001 | 4.68 | 0.048 | 4.67 | 0.001 | 4.59 | 0.000 | 4.67 | 0.238 | 4.74 | 0.215 | 4.73 | 0.170 |
| $S_{10}$ | 4.66 | 0.164 | 4.73 | 0.100 | 4.69 | 0.377 | 4.71 | 0.364 | 4.86 | 0.150 | 4.90 | 0.152 | 4.94 | 0.149 |

**Table S7.** Lowest energy absorption maxima of $P_r$ and $P_g$ from semiempirical methods. Wavelengths ($\lambda$), energies ($E_{max}$), energy differences in parenthesis and absolute absorption ($\varepsilon_{Pr}$ and $\varepsilon_{Pg}$) as well as photoproduct tuning ($\Delta E_{max}$) and ratio of absorption intensities ($\varepsilon_{Pg}/\varepsilon_{Pr}$) are tabulated. QM66 was employed and if not stated otherwise, the results are based on 10 snapshots taken every 100 ps and employing a cutoff of 12 Å to any of the QM atoms to take the environment as point charges into account.

| Method | $P_r$ | | | $P_g$ | | | Comparison | |
|---|---|---|---|---|---|---|---|---|
| | **λ (nm)** | **$E_{max}$ (eV)** | **$\varepsilon_{Pr}$[3]** | **λ (nm)** | **$E_{max}$ (eV)** | **$\varepsilon_{Pg}$[3]** | **$\Delta E_{max}$ (eV)** | **$\varepsilon_{Pg}/\varepsilon_{Pr}$** |
| **Exp.[1]** | 649 | 1.91 | - | 536 | 2.31 | - | 0.40 | 0.562 |
| **ZINDO/S** | | | | | | | | |
| 100 geom.[2] | 665 | 1.87 (-0.04) | 8.17 | 606 | 2.05 (-0.27) | 7.40 | 0.18 | 0.905 |
| 10 geom.[2] | 657 | 1.89 (-0.02) | 9.47 | 616 | 2.01 (-0.30) | 8.47 | 0.13 | 0.893 |
| 24 Å cutoff | 657 | 1.89 (-0.02) | 9.11 | 621 | 2.00 (-0.32) | 8.44 | 0.11 | 0.926 |
| **sTD-DFT** | | | | | | | | |
| 100 geom.[2] | 620 | 2.00 (+0.09) | 7.80 | 550 | 2.26 (-0.06) | 6.63 | 0.26 | 0.850 |
| 10 geom.[2] | 614 | 2.02 (+0.11) | 9.87 | 557 | 2.23 (-0.09) | 8.97 | 0.21 | 0.909 |
| 24 Å cutoff | 611 | 2.03 (+0.12) | 9.29 | 561 | 2.21 (-0.10) | 8.23 | 0.18 | 0.886 |
| sTDA | 572 | 2.17 (+0.26) | 15.01 | 527 | 2.35 (+0.04) | 12.90 | 0.18 | 0.859 |
| sTD-DFT (ωB97) | 530 | 2.34 (+0.43) | 11.68 | 492 | 2.52 (+0.21) | 9.93 | 0.18 | 0.850 |

[1] Taken from the spectra in Ref. [14] of the main text; [2] Values as reported in our previous publication, see Ref. [12] of the main text; [3] In units of $10^4$ L/(mol cm).

**Table S8.** Lowest energy absorption maxima of $P_r$ and $P_g$ employing a QM region consisting of 106 atoms. Wavelengths (λ), energies ($E_{max}$), energy differences in parenthesis and absolute absorption ($\varepsilon_{Pr}$ and $\varepsilon_{Pg}$) as well as photoproduct tuning ($\Delta E_{max}$) and ratio of absorption intensities ($\varepsilon_{Pg}/\varepsilon_{Pr}$) are tabulated. The cc-pVDZ basis set in case of the RI-ADC(2) and B3LYP calculations was employed. If not stated otherwise, the results are based on 10 snapshots taken every 100 ps and employing a cutoff of 12 Å to any of the QM atoms to take the environment as point charges into account.

| **Method** | **$P_r$** | | | **$P_g$** | | | **Comparison** | |
|---|---|---|---|---|---|---|---|---|
| | **λ (nm)** | **$E_{max}$ (eV)** | **$\varepsilon_{Pr}$ [3]** | **λ (nm)** | **$E_{max}$ (eV)** | **$\varepsilon_{Pg}$ [3]** | **$\Delta E_{max}$ (eV)** | **$\varepsilon_{Pg}/\varepsilon_{Pr}$** |
| **Exp.[1]** | 649 | 1.91 | - | 536 | 2.31 | - | 0.40 | 0.562 |
| **RI-ADC(2)** | | | | | | | | |
| 100 geom. [2] | 628 | 1.97 (+0.06) | 4.83 | 556 | 2.23 (-0.08) | 3.90 | 0.26 | 0.808 |
| 10 geom. [2] | 616 | 2.01 (+0.10) | 8.44 | 568 | 2.18 (-0.13) | 6.25 | 0.17 | 0.740 |
| Full Virtual Space[2] | 626 | 1.98 (+0.07) | 8.46 | 577 | 2.15 (-0.16) | 6.11 | 0.17 | 0.740 |
| **TD-DFT** | | | | | | | | |
| B3LYP | 586 | 2.12 (+0.21) | 8.88 | 567 | 2.19 (-0.13) | 6.04 | 0.07 | 0.680 |
| **ZINDO/S** | | | | | | | | |
| 100 geom. [2] | 677 | 1.83 (-0.08) | 7.63 | 608 | 2.04 (-0.27) | 6.50 | 0.21 | 0.852 |
| 10 geom. [2] | 673 | 1.84 (-0.07) | 8.72 | 624 | 1.99 (-0.33) | 6.85 | 0.14 | 0.785 |
| 24 Å cutoff | 672 | 1.85 (-0.07) | 8.69 | 622 | 1.99 (-0.32) | 7.33 | 0.15 | 0.844 |
| **sTD-DFT** | | | | | | | | |
| 100 geom. [2] | 627 | 1.98 (+0.07) | 7.02 | 553 | 2.24 (-0.07) | 5.78 | 0.27 | 0.823 |
| 10 geom. [2] | 621 | 2.00 (+0.09) | 8.07 | 571 | 2.17 (-0.14) | 6.80 | 0.18 | 0.843 |
| 24 Å cutoff | 621 | 2.00 (+0.09) | 8.41 | 563 | 2.20 (-0.11) | 7.28 | 0.20 | 0.865 |
| sTDA | 580 | 2.14 (+0.23) | 12.71 | 540 | 2.30 (-0.02) | 9.95 | 0.16 | 0.783 |
| sTD-DFT (ωB97) | 544 | 2.28 (+0.37) | 9.87 | 506 | 2.45 (+0.14) | 8.66 | 0.17 | 0.878 |

[1] Taken from the spectra in Ref. [14] of the main text; [2] Values as reported in our previous publication, see Ref. [12] of the main text; [3] In units of $10^4$ L/(mol cm).

**Table S9.** Lowest energy absorption maxima of $P_r$ and $P_g$ from multi-reference calculations. Wavelengths (λ), energies ($E_{max}$), energy differences in parenthesis and absolute absorption ($\varepsilon_{Pr}$ and $\varepsilon_{Pg}$) as well as photoproduct tuning ($\Delta E_{max}$) and ratio of absorption intensities ($\varepsilon_{Pg}/\varepsilon_{Pr}$) are tabulated. In all cases, the QM region consisted of 66 atoms and the results are based on 10 snapshots taken every 100 ps and employing a cutoff of 12 Å to any of the QM atoms to take the environment as point charges into account.

| **Method** | **$P_r$** | | | **$P_g$** | | | **Comparison** | |
|---|---|---|---|---|---|---|---|---|
| | **λ (nm)** | **$E_{max}$ (eV)** | **$\varepsilon_{Pr}$ [2]** | **λ (nm)** | **$E_{max}$ (eV)** | **$\varepsilon_{Pg}$ [2]** | **$\Delta E_{max}$ (eV)** | **$\varepsilon_{Pg}/\varepsilon_{Pr}$** |
| **Exp.[1]** | 649 | 1.91 | - | 536 | 2.31 | - | 0.40 | 0.562 |
| **NEVPT(2)** | | | | | | | | |
| PC (20/13) | 588 | 2.11 (+0.20) | 7.17 | 569 | 2.18 (-0.13) | 7.75 | 0.07 | 1.082 |
| SC (20/13) | 556 | 2.23 (+0.32) | 7.91 | 536 | 2.31 (+0.00) | 8.39 | 0.08 | 1.060 |
| **OM2-MRCISD** | | | | | | | | |
| (40/40) | 580 | 2.14 (+0.23) | 9.33 | 537 | 2.31 (+0.00) | 9.55 | 0.17 | 1.023 |
| (20/20) | 550 | 2.25 (+0.34) | 10.58 | 510 | 2.43 (+0.12) | 10.31 | 0.18 | 0.974 |
| (20/20) + Triples | 570 | 2.17 (+0.26) | 9.49 | 526 | 2.36 (+0.04) | 9.36 | 0.18 | 0.986 |

[1] Taken from the spectra in Ref. [14] of the main text; [2] In units of $10^4$ L/(mol cm).

**Table S10.** Root mean square deviations of geometries in Å. Comparisons are given for methods in the corresponding column and row for $P_r$ (first value) and $P_g$ (second value, shown in parenthesis). For alignment, the geometries were reduced to 42 atoms as in case of the optimizations *in vacuo*. They were obtained from RI-BLYP+D3/AMBER optimized structures, where the QM region either consisted of 106 atoms (QM106) or 66 atoms (QM66). In case of the latter, optimizations were not only performed in cartesian coordinates, but also in hybrid delocalized coordinates (HDLC).

| $P_r$ ($P_g$) | QM66 | QM66 HDLC |
|---|---|---|
| **QM106** | 0.08 (0.08) | 0.08 (0.07) |
| **QM66** | - | 0.03 (0.03) |

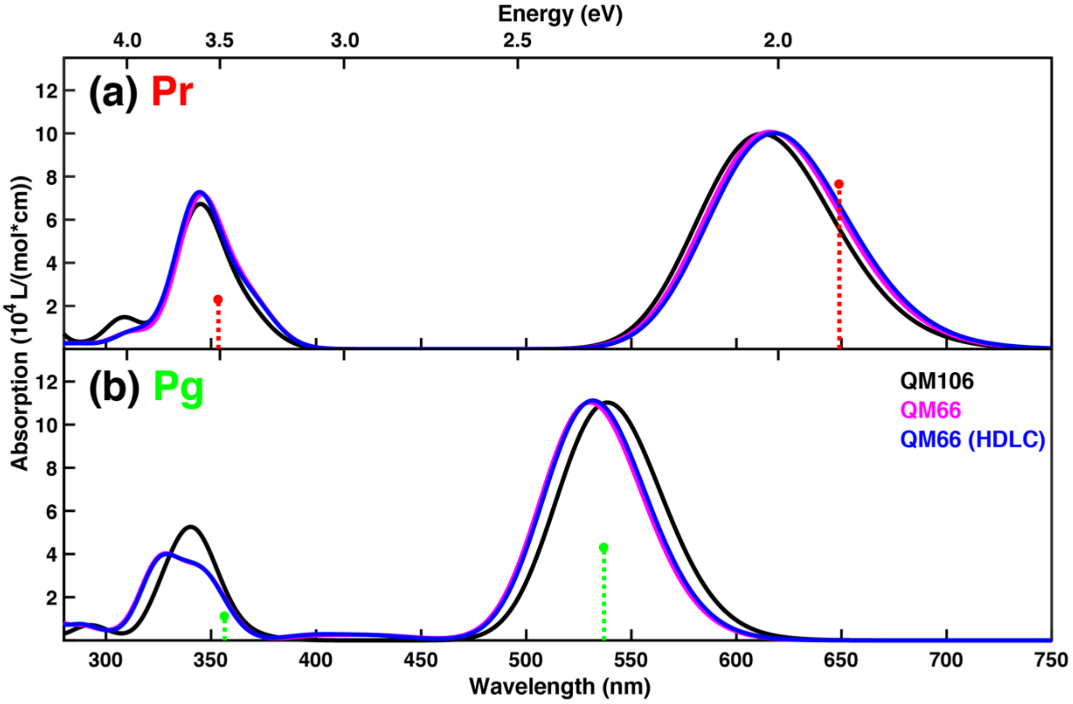


**Figure S1.** (**a**) Absorption spectra for RI-BLYP+D3/AMBER optimized structures with QM66 or QM106 (black) from subsequent sTD-DFT calculations for the $P_r$ form employing QM106 for the excited state calculations. In case of the smaller QM region for optimization, either cartesian (magenta) or hybrid delocalized coordinates (HDLC, blue) were used; (**b**) Absorption spectra for RI-BLYP+D3/AMBER optimized structures from subsequent sTD-DFT calculations for the $P_g$ form analogous to the results from $P_r$.

**Table S11.** Lowest energy absorption maxima of $P_r$ and $P_g$ from sTD-DFT calculations for RI-BLYP+D3/AMBER optimized structures. Wavelengths (λ), energies ($E_{max}$), energy differences in parenthesis and absolute absorption ($\varepsilon_{Pr}$ and $\varepsilon_{Pg}$) as well as photoproduct tuning ($\Delta E_{max}$) and ratio of absorption intensities ($\varepsilon_{Pg}/\varepsilon_{Pr}$) are tabulated.. In all cases, the QM region for excited state calculations consisted of 106 atoms and the values are extracted from the spectra in Figure S1.

| Method | $P_r$ | | | $P_g$ | | | Comparison | |
|---|---|---|---|---|---|---|---|---|
| | λ (nm) | $E_{max}$ (eV) | $\varepsilon_{Pr}$[2] | λ (nm) | $E_{max}$ (eV) | $\varepsilon_{Pg}$[2] | $\Delta E_{max}$ (eV) | $\varepsilon_{Pg}/\varepsilon_{Pr}$ |
| **Exp.**[1] | 649 | 1.91 | - | 536 | 2.31 | - | 0.40 | 0.562 |
| QM106 | 612 | 2.03 (+0.12) | 9.98 | 539 | 2.30 (-0.01) | 11.02 | 0.28 | 1.105 |
| QM66 | 616 | 2.01 (+0.10) | 10.07 | 531 | 2.34 (+0.02) | 11.02 | 0.32 | 1.094 |
| QM66 + HDLC | 618 | 2.01 (+0.10) | 10.01 | 532 | 2.33 (+0.02) | 11.11 | 0.33 | 1.110 |

[1] Taken from the spectra in Ref. [14] of the main text; [2] In units of $10^4$ L/(mol cm).

**Table S12.** Root mean square deviations of geometries in Å. Comparisons are given for methods in the corresponding column and row for $P_r$ (first value) and $P_g$ (second value, in parenthesis). For alignment, the geometries were reduced to 42 atoms as in the case of the optimizations *in vacuo*. They were obtained from DFTB2+D/AMBER optimized structures with QM66. The structures denoted as "Initial" were obtained from 100,000 steps of steepest descent (SD) optimizations. Taking the final structure as starting point, up to 100,000 steps of further optimizations were performed with the conjugate gradient (CG), SD and a limited-memory Broyden-Fletcher-Goldfarb-Shanno algorithm implemented in XMIN (AMBER software package). However, for CG and XMIN the optimizations ended before reaching the maximum number of iterations. In addition, the DFTB2+D/AMBER optimizations results for 100,000 steps of SD starting from the RI-BLYP+D3/AMBER optimized structure are also shown (from BLYP).

| $P_r$ ($P_g$) | CG | SD | XMIN | From BLYP |
|---|---|---|---|---|
| **Initial** | 0.01 (0.01) | 0.00 (0.00) | 0.01 (0.02) | 0.04 (0.05) |
| **CG** | - | 0.00 (0.01) | 0.01 (0.01) | 0.05 (0.05) |
| **SD** | | - | 0.01 (0.02) | 0.04 (0.05) |
| **XMIN** | | | - | 0.05 (0.06) |

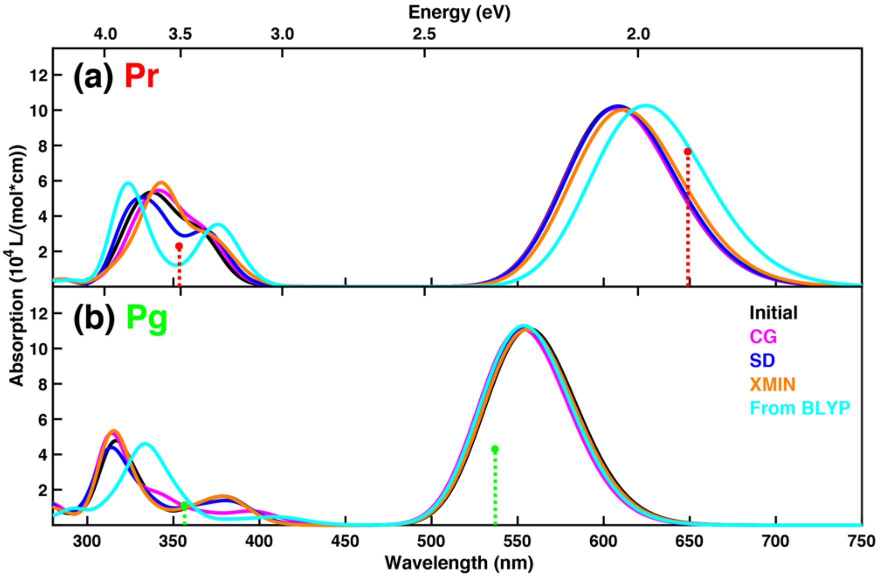


**Figure S2.** (**a**) Absorption spectra for DFTB2+D/AMBER optimized structures from subsequent sTD-DFT calculations for the $P_r$ form employing 106 atoms. Shown are the spectra from structures of an initial 100,000 step steepest descent optimization (Initial, black), of subsequent optimizations employing up to 100,000 steps with conjugate gradient (CG, magenta), steepest descent (SD, blue), and a limited-memory Broyden-Fletcher-Goldfarb-Shanno algorithm implemented in XMIN (orange). In addition, the spectrum obtained from 100,000 steps of SD starting with the RI-BLYP+D3/AMBER optimized structure is also shown (From BLYP, cyan) and all optimizations were performed with QM66. (**b**) Absorption spectra for DFTB2+D/AMBER optimized structures from subsequent sTD-DFT calculations for the $P_g$ form analogous to the results from $P_r$.

**Table S13.** Lowest energy absorption maxima of $P_r$ and $P_g$ from sTD-DFT calculations for DFTB2+D/AMBER optimized structures. Wavelengths ($\lambda$), energies ($E_{max}$), energy differences in parenthesis and absolute absorption ($\varepsilon_{Pr}$ and $\varepsilon_{Pg}$) as well as photoproduct tuning ($\Delta E_{max}$) and ratio of absorption intensities ($\varepsilon_{Pg}/\varepsilon_{Pr}$) are tabulated. In all cases, QM66 was employed for optimizations and QM106 for excited state calculations. The values are extracted from the spectra in Figure S2.

| Method | $P_r$ | | | $P_g$ | | | Comparison | |
|---|---|---|---|---|---|---|---|---|
| | $\lambda$ (nm) | $E_{max}$ (eV) | $\varepsilon_{Pr}$ [2] | $\lambda$ (nm) | $E_{max}$ (eV) | $\varepsilon_{Pg}$ [2] | $\Delta E_{max}$ (eV) | $\varepsilon_{Pg}/\varepsilon_{Pr}$ |
| **Exp.** [1] | 649 | 1.91 | - | 536 | 2.31 | - | 0.40 | 0.562 |
| Initial | 607 | 2.04 (+0.13) | 10.17 | 557 | 2.23 (-0.09) | 11.13 | 0.19 | 1.094 |
| CG | 607 | 2.04 (+0.13) | 10.12 | 552 | 2.25 (-0.07) | 11.14 | 0.20 | 1.101 |
| SD | 608 | 2.04 (+0.13) | 10.23 | 554 | 2.24 (-0.08) | 11.18 | 0.20 | 1.093 |
| XMIN | 612 | 2.03 (+0.12) | 10.02 | 556 | 2.23 (-0.08) | 11.07 | 0.20 | 1.105 |
| From BLYP | 624 | 1.99 (+0.08) | 10.26 | 553 | 2.24 (-0.07) | 11.29 | 0.25 | 1.100 |

[1] Taken from the spectra in Ref. [14] of the main text; [2] In units of $10^4$ L/(mol cm).

**Table S14.** Lowest energy absorption maxima of $P_r$ and $P_g$ from sTD-DFT calculations for (QM/)MM optimized structures. Wavelengths ($\lambda$), energies ($E_{max}$), energy differences in parenthesis and absolute absorption ($\varepsilon_{Pr}$ and $\varepsilon_{Pg}$) as well as photoproduct tuning ($\Delta E_{max}$) and ratio of absorption intensities ($\varepsilon_{Pg}/\varepsilon_{Pr}$) are tabulated. For QM/MM optimizations, QM66 was employed and QM106 was used in all cases for excited state calculations. The values are extracted from the spectra in Figure 3.

| Method | $P_r$ | | | $P_g$ | | | Comparison | |
|---|---|---|---|---|---|---|---|---|
| | λ (nm) | $E_{max}$ (eV) | $\varepsilon_{Pr}$[2] | λ (nm) | $E_{max}$ (eV) | $\varepsilon_{Pg}$[2] | $\Delta E_{max}$ (eV) | $\varepsilon_{Pg}/\varepsilon_{Pr}$ |
| **Exp.**[1] | 649 | 1.91 | - | 536 | 2.31 | - | 0.40 | 0.562 |
| RI-BLYP+D3 | 618 | 2.01 (+0.10) | 10.01 | 532 | 2.33 (+0.02) | 11.11 | 0.33 | 1.110 |
| RI-MP2 | 600 | 2.07 (+0.16) | 10.04 | 504 | 2.46 (+0.15) | 10.92 | 0.40 | 1.088 |
| RI-CC2 | 618 | 2.01 (+0.10) | 9.73 | 511 | 2.43 (+0.11) | 10.90 | 0.42 | 1.121 |
| AMBER | 703 | 1.76 (-0.15) | 9.13 | 633 | 1.96 (-0.35) | 9.97 | 0.19 | 1.092 |
| DFTB2+D | 607 | 2.04 (+0.13) | 10.17 | 557 | 2.23 (-0.09) | 11.13 | 0.19 | 1.094 |

[1] Taken from the spectra in Ref. [14] of the main text; [2] In units of $10^4$ L/(mol cm).

**Table S15.** Arithmetic mean values of the excitation energies (E), oscillator strengths (f) and root mean square electron-hole separation ($RMS_{eh}$) for $P_r$ and $P_g$ as well as their differences obtained from wave function analysis for the lowest excited state with the exception of the BLYP calculations. In all cases, the QM region consisted of 66 atoms and if not stated otherwise, the results are based on 10 snapshots taken every 100 ps, the cc-pVDZ basis set was utilized, and a cutoff of 12 Å to any of the QM atoms was employed to take the environment as point charges into account.

| Method | $P_r$ | | | $P_g$ | | | Difference | | |
|---|---|---|---|---|---|---|---|---|---|
| | E (eV) | f | $RMS_{eh}$ | E (eV) | f | $RMS_{eh}$ | ΔE (eV) | Δf | $\Delta RMS_{eh}$ |
| **RI-ADC(2)** | | | | | | | | | |
| 100 geom. [1] | 2.040 | 1.049 | 5.108 | 2.272 | 1.040 | 4.813 | 0.231 | -0.010 | -0.295 |
| 10 geom.[1] | 2.066 | 1.064 | 5.109 | 2.204 | 1.015 | 4.909 | 0.138 | -0.049 | -0.200 |
| 24 Å cutoff | 2.075 | 1.075 | 5.049 | 2.205 | 1.027 | 4.869 | 0.131 | -0.048 | -0.180 |
| cc-aug-pVDZ | 2.014 | 1.016 | 5.233 | 2.144 | 0.955 | 4.998 | 0.130 | -0.061 | -0.235 |
| **WF-based** | | | | | | | | | |
| RI-CC2 | 2.215 | 1.295 | 5.147 | 2.354 | 1.210 | 4.997 | 0.139 | -0.085 | -0.150 |
| RI-CCS | 2.757 | 1.557 | 4.681 | 2.932 | 1.529 | 4.357 | 0.175 | -0.028 | -0.324 |
| CIS | 2.758 | 1.581 | 4.277 | 2.933 | 1.541 | 3.996 | 0.175 | -0.039 | -0.282 |
| TD-HF | 2.506 | 1.409 | 4.221 | 2.681 | 1.389 | 3.938 | 0.175 | -0.020 | -0.283 |
| **DFT-based** | | | | | | | | | |
| CAM-B3LYP | 2.304 | 1.196 | 5.056 | 2.449 | 1.143 | 4.814 | 0.146 | -0.053 | -0.242 |
| B3LYP | 2.179 | 1.077 | 5.902 | 2.241 | 0.911 | 5.772 | 0.062 | -0.166 | -0.129 |
| B3LYP (TDA) | 2.468 | 1.681 | 6.160 | 2.459 | 1.272 | 5.990 | -0.008 | -0.410 | -0.170 |
| BLYP[2] | 2.030 | 0.840 | 6.575 | 1.974 | 0.618 | 6.632 | -0.056 | -0.221 | 0.057 |

[1] Values from calculations reported in our previous study, see Ref. [12] of the main text; [2] For the statistics, we have considered excitations that are dominated by a transition from HOMO to LUMO. Owing to this and in case of BLYP, for 3 snapshots of $P_r$ and 1 snapshot of $P_g$ the $S_2$ state was considered, as in those cases the $S_1$ state was dominated by a charge transfer excitation from HOMO-1 to LUMO.

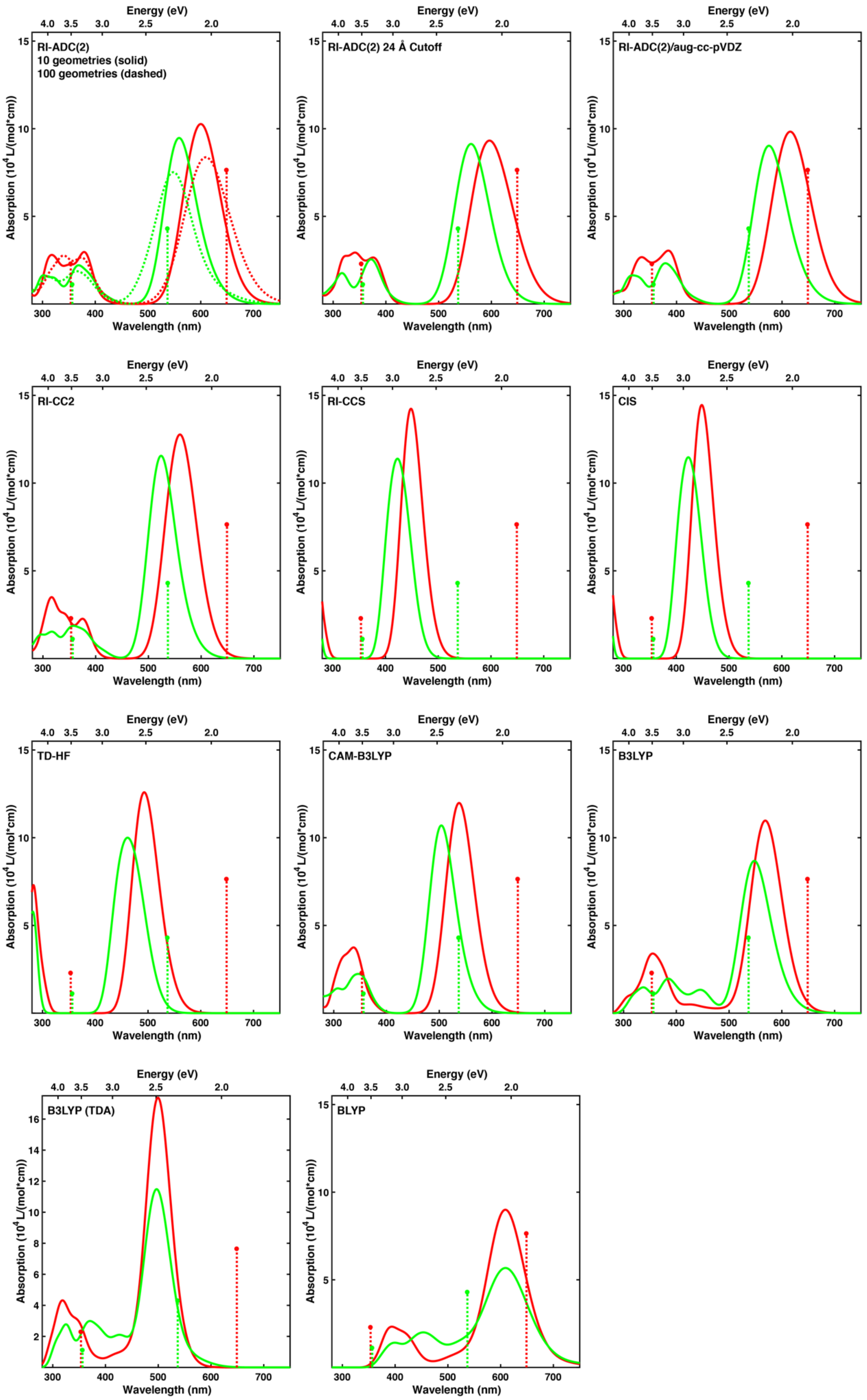


**Figure S3:** Spectra from which the values presented in Table 2 were derived and the corresponding method is indicated with an inset. For details of the computations, see the main text.

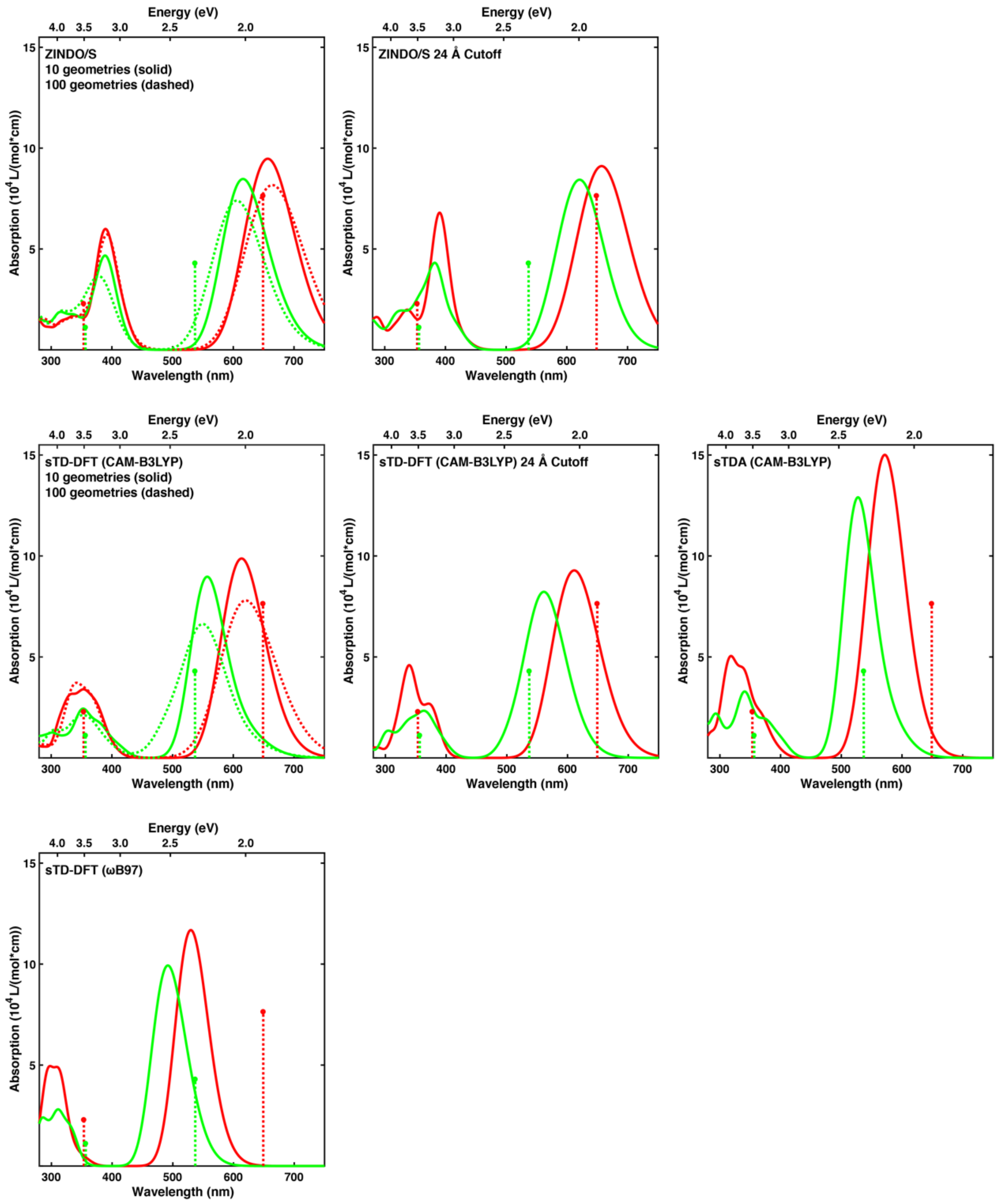


**Figure S4:** Spectra from which the values presented in Table S7 were derived and the corresponding method is indicated with an inset. For details of the computations, see the main text.

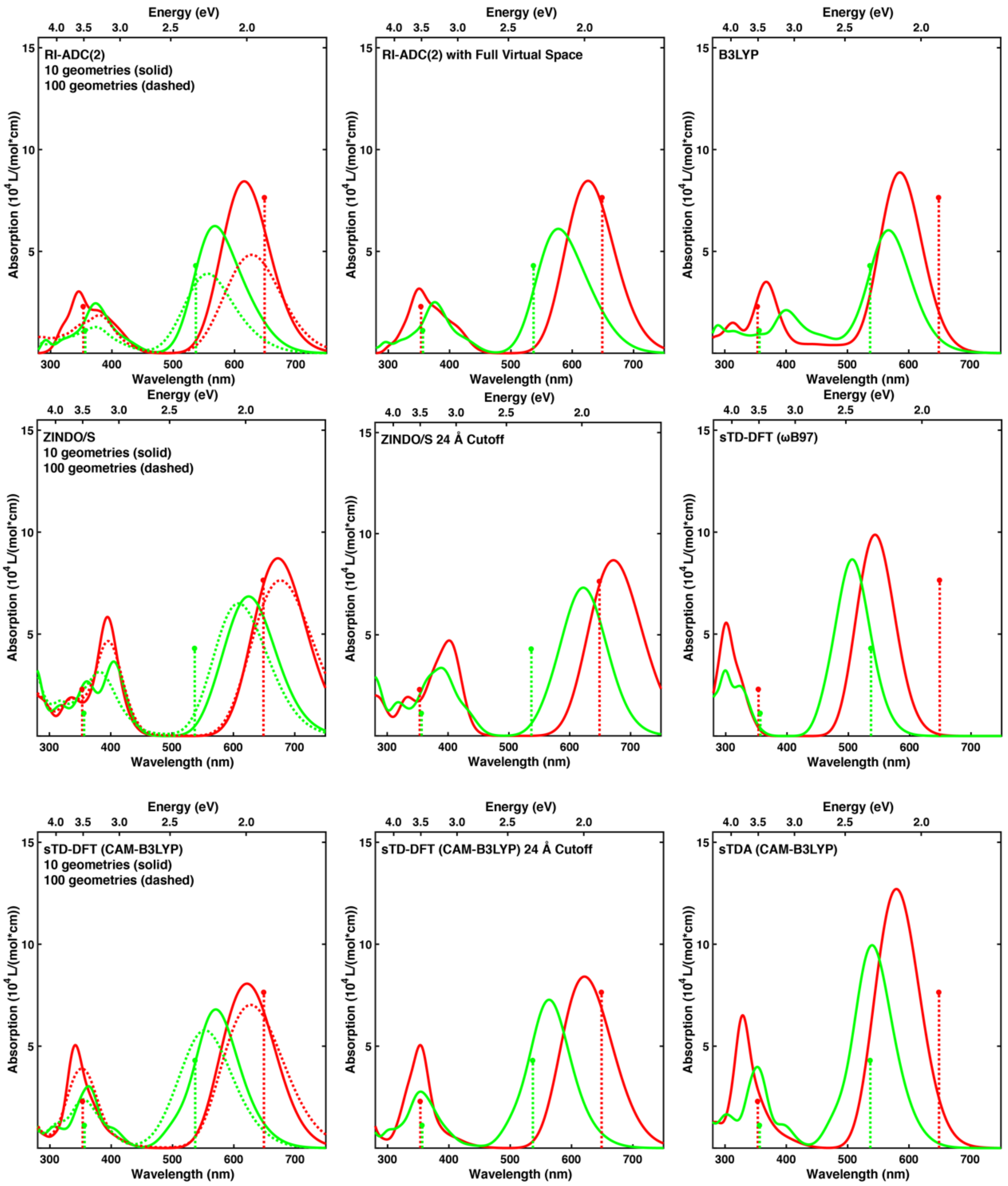


**Figure S5:** Spectra from which the values presented in Table S8 were derived, *i.e.* excited state calculations with QM106, and the corresponding method is indicated with an inset. For details of the computations, see the main text.

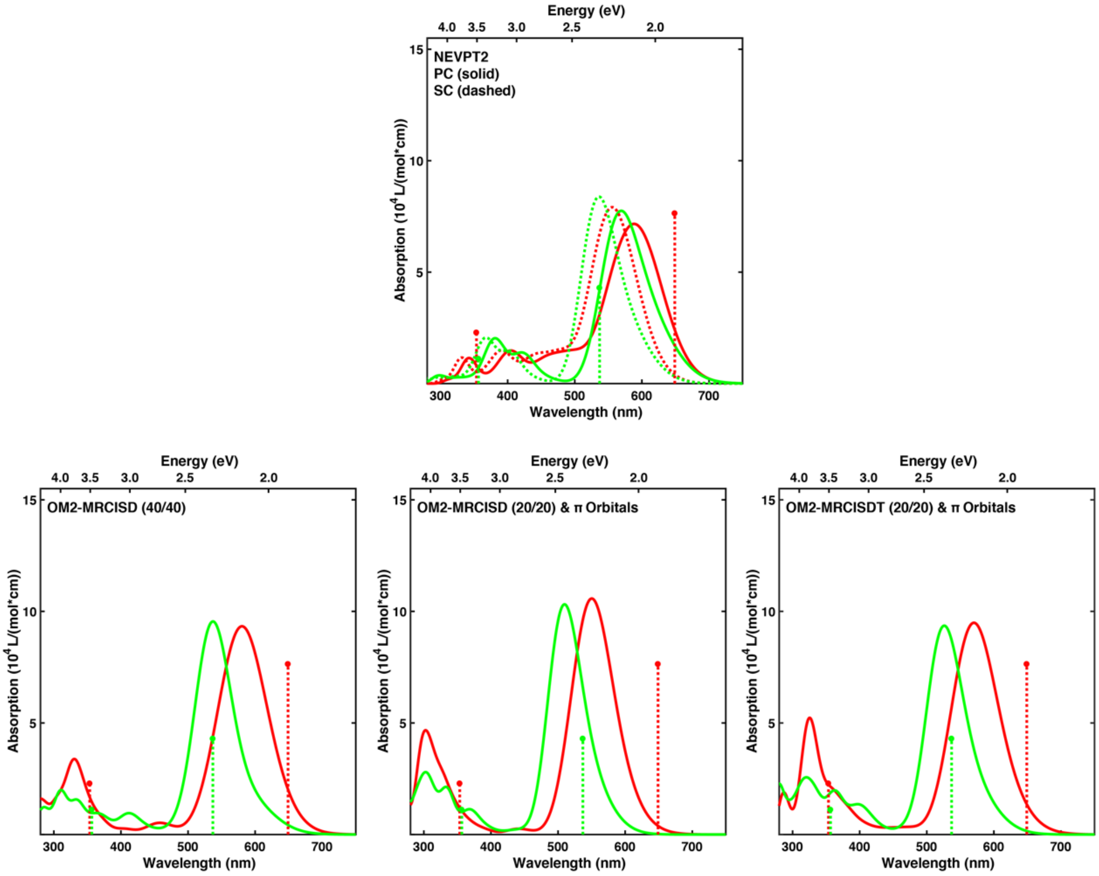


**Figure S6:** Spectra from which the values presented in Table S9 were derived and the corresponding method is indicated with an inset. For details of the computations, see the main text. Note that in case of the NEVPT2 calculations, the 5 excited states considered are not sufficient to cover the second absorption band.
